# DNA Methylation Reflects Cis‐Genetic Differentiation Across the European Crow Hybrid Zone

**DOI:** 10.1111/mec.70026

**Published:** 2025-07-10

**Authors:** Justin Merondun, Jochen B. W. Wolf

**Affiliations:** ^1^ Division of Evolutionary Biology LMU Munich Martinsried Germany; ^2^ Microevolution and Biodiversity Max Planck Institute for Biological Intelligence Seewiesen Germany; ^3^ Department of Biology University of Victoria Victoria British Columbia Canada

**Keywords:** DNA methylation, evolutionary epigenetics, genomics, hybridization, speciation

## Abstract

Chromatin modifications provide a substrate for epigenetic variation with evolutionary potential. To quantify the contribution of this layer of variation during speciation in crows, we leveraged genome and methylome sequencing data from an incipient avian species: all‐black carrion crows, grey‐coated hooded crows, and their hybrids. Combining controlled experimentation under common garden conditions and sampling of natural genetic variation across the hybrid zone, we show that 5mC methylation variation was largely explained by genome properties and the ontogenetic programme of the organism. Taxonomically related methylation divergence clustered in intergenic space, with the only genomic region of strongly elevated genetic differentiation encoding the diagnostic colour contrast between taxa. We conclude that methylation variation with relevance to speciation largely follows *cis*‐genetic polymorphism in this system and does not constitute an autonomous axis of evolution.

## 1 Introduction

Mutations are the ultimate source of evolution. Prevailing evolutionary theory conceptualises mutations as random changes to the DNA backbone filtered by selection, depleted by genetic drift, reorganised by recombination, and redistributed by migration (Wright 1931). The concept of epigenetic inheritance challenges this paradigm, as it introduces a second layer of potentially heritable variation that is not subject to alterations of the nucleotide sequence (Jablonka and Raz 2009). DNA methylation is a prime candidate of a molecular epigenetic inheritance system featuring variation along the genome, within and among individuals and populations (Fitz‐James and Cavalli 2022; Heard and Martienssen 2014). The underpinnings of variation in DNA methylation are diverse and include (i) spontaneous epimutations, (ii) environmental induction, (iii) physiological processes establishing somatic cell fate (ontogenetic programme sensu Waddington 2008) and (iv) genetic constraints imposed by genome properties (chromosomal or genomic features) or the organism's genotype (Taudt et al. 2016). While the latter two sources are readily incorporated into traditional evolutionary theory, the first two are not. To judge the autonomous potential of DNA methylation for evolution, it is therefore crucial to obtain a quantitative understanding of the sources shaping natural variation (Christina and Pigliucci 2020; Mueller et al. 2025).

In plants, evolutionarily relevant methylation variation seems rather widespread (Zhang et al. 2018). There is evidence for spontaneous, random epimutations in DNA methylation (Hazarika et al. 2022; van der Graaf et al. 2015; Yao et al. 2021), environmentally induced epimutations (Kawakatsu et al. 2016) and transgenerational inheritance of both (Cubas et al. 1999; Furci et al. 2019; Manning et al. 2006). In animals, with strict soma‐germline separation and epigenetic reprogramming (Reik et al. 2001), variation in 5mC methylation that is independent of the genotype is expected to be more limited (Horsthemke 2018). While animal research aiming to separate genetic, developmental, and environmental impacts on variation in DNA methylation is gaining traction (Beck et al. 2021), the evidence for stable inheritance of autonomous chromatin modifications remains scarce (Beck et al. 2021; Leroux et al. 2017; Mueller et al. 2025; Pierron et al. 2021; Thorson et al. 2021). Numerous vertebrate studies have linked 5mC methylation variation with environmental change (Le Luyer et al. 2017; Meröndun et al. 2019; Wang et al. 2022) and/or phenotypic variation (Cossette et al. 2023), but few have done so while controlling for confounding genetic effects (Mueller et al. 2025) (see also (Laporte et al. 2019) for transposable elements). In birds, DNA methylation has been associated with stress resilience in tree swallows (Taff et al. 2019) and urbanisation in great tits (Watson et al. 2021), while conversely a recent well‐designed study employing partial cross‐fostering under controlled conditions suggests a very limited role for environmental induction on methylation variation independent of the genotype (Sepers et al. 2023). Additional avian research in house sparrows suggests a role for changes in epigenetic potential (i.e., the number of CpG motifs in the genome) during range expansions, while DNA methylation variation itself was inconsequential (Hanson et al. 2022).

Hybrid zones are suitable natural models to decompose heritable genetic and epigenetic variation of diverged populations. In the central part of the hybrid zone, environmental variation is limited while genetic variation is maximised in mosaic hybrid genomes that are characterised by blocks of alternating ancestry. These properties have been successfully exploited to map the genetic basis of phenotypic variation and advance our understanding of the processes governing population divergence (Barton and Hewitt 1985; Gompert et al. 2017; Knief et al. 2019). For studies of epigenetic variation, hybrid zones confer similar advantages (Baldassarre et al. 2014). We here made use of this fact and quantified both genetic and methylation variation in a well‐studied avian hybrid zone.

All‐black carrion crows (*Corvus* (*corone*) *corone*) and grey‐coated hooded crows (*C*. (*c*.) *cornix*) hybridise in a narrow contact zone in central Europe, which is governed by assortative mating and social dynamics related to plumage pigmentation patterns (Metzler et al. 2021). Genome scans have found minimal genetic divergence across most of the genome, which is homogenised by high levels of gene flow following secondary contact (Gwee et al. 2025). A notable exception is a ~2‐Mb region on chromosome 18, hereafter referred to as the *focal region*, which is subject to divergent selection, has accumulated fixed differences between parental taxa, and is mainly responsible for the striking phenotypic variation (Knief et al. 2019; Poelstra et al. 2014; Vijay et al. 2016; Weissensteiner et al. 2020). Recombination is reduced within this *focal region* in both ancestries, preserving linkage disequilibrium of ancestral genetic variation (Knief et al. 2019; Weissensteiner et al. 2017). The genetic heterogeneity of the system, where a largely homogenous genome‐wide background is opposed to a diverged *focal region* known to be relevant for speciation, provides a unique opportunity to gain insight into the determinants of DNA methylation variation within and between taxa.

## 2 Materials and Methods

### 2.1 Overview of Study Design

**FIGURE 1:**
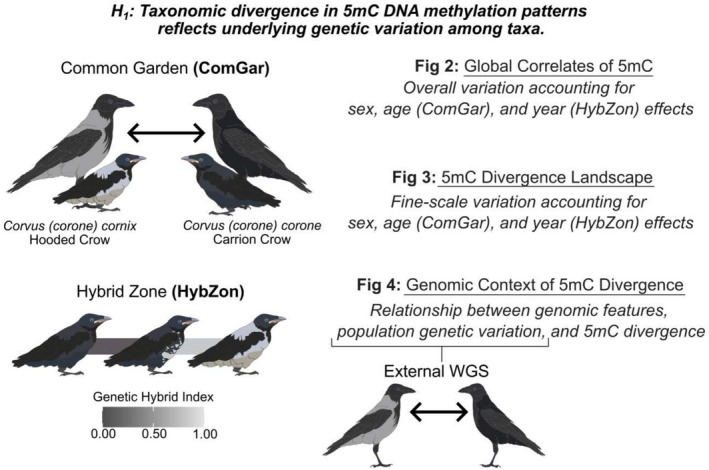
Sampling and analytical strategy to assess DNA methylation variation in crows. DNA methylation patterns were assessed in blood tissue across two experimental contexts: A common garden (ComGar) design in which grey‐coated hooded crows (*Corvus* (*corone*) *cornix*) and black‐coated carrion crows (*Corvus* (*corone*) *corone*) were reared under standardised environmental conditions with balanced sex representation through adulthood across three developmental stages (Chick, Yearling, Adult); and a hybrid zone (HybZon) transect design in which male nestlings were sampled along a gradient of genomic admixture from hooded to carrion crow ancestry. Taxonomic 5mC divergence was quantified using (i) multivariate ordinations (Fig 2), (ii) fine‐scale identification of differentially methylated sites (Fig 3), and (iii) machine learning models incorporating measures of population genetic variation and genomic context (Fig 4) to evaluate the determinants of methylation patterning. ComGar, common garden; HybZon, hybrid zone; WGS, whole genome sequencing.

**FIGURE 2:**
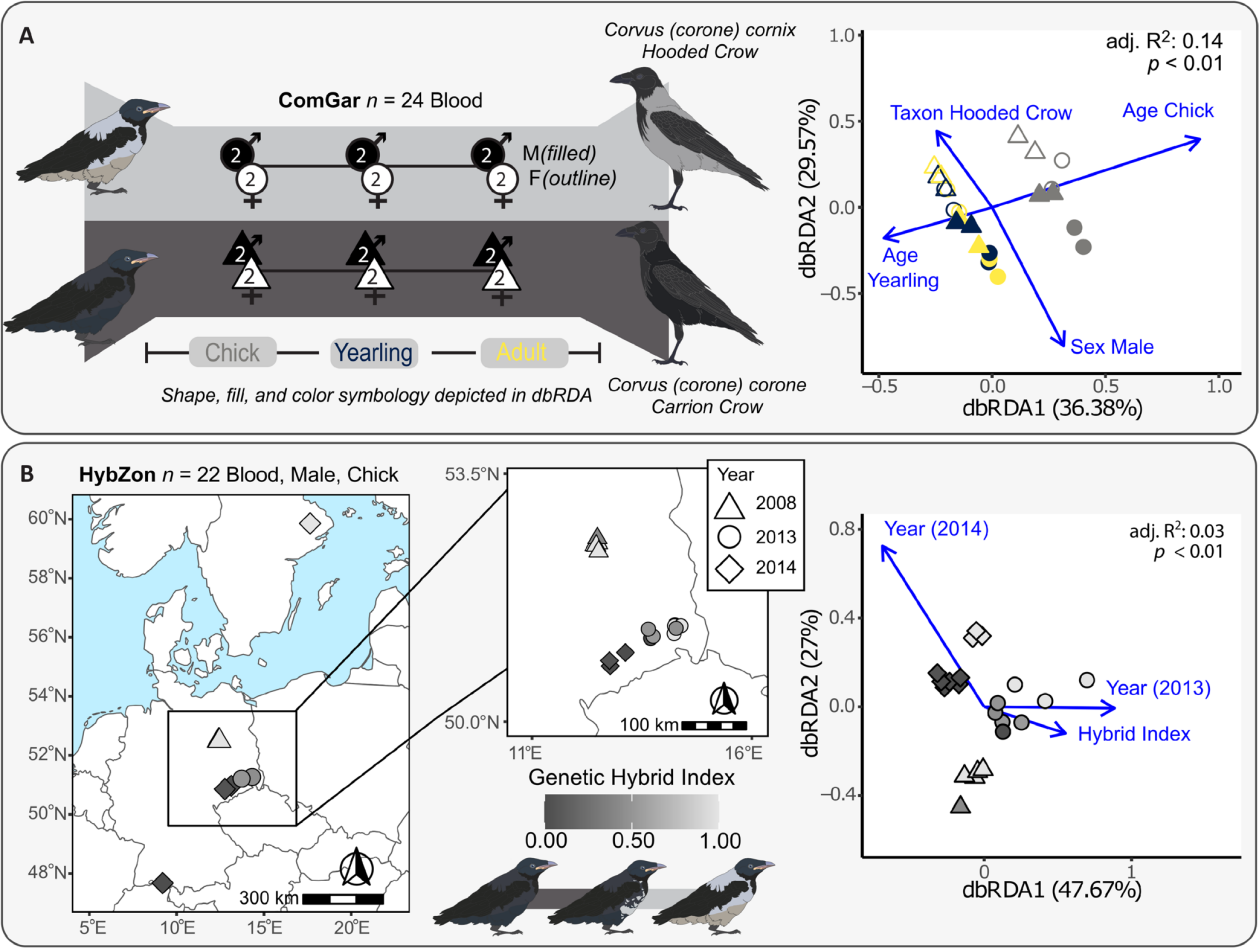
Detailed sampling design and global DNA methylation correlates. Left panels illustrate the sampling frameworks for the common garden (ComGar) and hybrid zone transect (HybZon) designs. Right panels display distance‐based redundancy analyses (dbRDA) summarising genome‐wide DNA methylation structure for each experiment. (A) In ComGar, RRBS data isolate effects of age, sex, and taxon under controlled environmental conditions (1.05 M CpGs post‐filtering). (B) In HybZon, RRBS data capture methylation variation across a gradient of genetic ancestry and sampling years, by design controlling for tissue type, sex, and age (1.40 M CpGs). Adj. *R* ^2^, adjusted pseudo‐*R* ^2^; ComGar, common garden; HybZon, hybrid zone; *p*, *p*‐value from dbRDA.

We pursued two sampling strategies (Figure 1). We raised wild‐caught nestlings from pure parental populations in Germany and Sweden to adulthood in a common garden experiment (hereafter ComGar, Figure 2A) and sampled additional nestlings across a genetic hybrid zone transect in Central Europe (hereafter HybZon; Figure 2B). The ComGar was primarily designed to establish a baseline of physiological determinants of 5mC methylation (age, sex) and test for taxon differences under controlled conditions; whereas the HybZon experiment is suited to study how signatures of DNA methylation covary with genetic ancestry across hybrid individuals. In conjunction with previously generated whole‐genome resequencing data, both approaches further address the effects of genome properties (chromosomal and genomic features) and genetic variation within and between taxa (*D*_XY_, *F*_ST_, haplotype diversity, Tajima's *D*, Fu & Li's D*). To quantify the intensity of 5mC methylation per CpG site, we generated reduced representation bisulfite sequencing (RRBS) data which allow for comparisons of orthologous sites with high read coverage between individuals (ComGar mean and standard deviation CpG coverage: 31.2 ± 3.87×; HybZon: 45.2 ± 4.88×; Table S1). After filtering for CpGs supported by a minimum of 10 reads and less than 10% missingness across individuals within each experiment, we retained a total of 1,051,004 (ComGar) and 1,415,294 (HybZon) high‐quality CpG sites. In these RRBS libraries, which are highly enriched for promoter regions, nearly half of the assayed CpGs were invariable across all individuals (abs (max_5mC_—min_5mC_) < 10%; 48.5% in ComGar, 49.6% in HybZon).

We used two approaches to assess the contributions of candidate variables (age, sex, taxon) to methylation patterning (Figure 1): multivariate statistics examining global correlates of methylation variation and differential methylation analysis allowing for base‐pair resolution of single differentially methylated positions (DMPs), requiring a stringent minimum effect size between groups (minimum 25% mean proportion difference and FDR‐corrected *p* < 0.01). Capitalising on the role of methylation in speciation, we further leveraged our multi‐experimental design to detect DMPs which are significantly associated with taxon across both sampling experiments. While allowing a margin for false negatives, this approach reduces spurious effects and provides a perspective on multi‐experimental validation across sequencing methods and biological replicates, which are rarely controlled for in studies of methylation variation (Mueller et al. 2025; Sepers et al. 2019). The aim here was not to find as many taxon‐related DMPs as possible, but to reduce false positives to a minimum and identify the DMPs with the largest effect sizes.

### 2.2 Sampling—Common Garden

For an illustrative overview of the sampling design see Figure 1. We designed the ComGar experiment to quantify DNA methylation variation corresponding to physiological factors (age, sex) and taxon differences. We first collected blood from the brachial vein of unrelated nestlings with a mean age of 22.1 days (range 14–26) from purebred carrion (*n* = 4) and hooded crows (*n* = 4). Carrion crows were collected in May 2014 from different nests in Konstanz in Southern Germany (47°45 N′, 9°10′E) as part of a larger research programme. Hooded crows were sampled around Uppsala, Sweden (59°52′N, 17°38′E) in the same month and year. These 8 individuals were transferred to Sweden by airplane and hand‐raised indoors at Tovetorp field station, Sweden (58°56′N, 17°8′E). At the time when birds could feed independently, they were released into a roofed outdoor enclosure (6.5 × 4.8 × 3.5 m) and thereafter housed in single‐sex groups of the same species under common garden conditions. For details of animal husbandry see (Holtmann et al. 2019). Blood sampling was repeated for these 8 individuals during the non‐reproductive season at an age of 18 months (551–565 days) and during the reproductive season at an age of 30 months when sexual maturity is expected to have been reached in all individuals (916–930 days) (von Blotzheim et al. 1993). For this common garden experiment, sample sizes of both carrion and hooded crows raised in captivity were matched by sex (2 males and 2 females of each taxon) which was determined molecularly (Griffith et al. 1998) (Table S1). The 24 blood samples—8 individuals at three time points—were subjected to RRBS to quantify sex, age, and taxon differences. Furthermore, we corroborated all analyses and identified the effects of tissue variation on methylation patterns with whole‐genome bisulfite sequencing (WGBS) data for a subset of four fully mature individuals from the same common garden (two *C*. (*c*.) *cornix*, two *C*. (*c*.) *corone*, one male and one female per taxon), each sampled across three tissues (blood, liver, spleen) (Data S1).

### 2.3 Sampling—Hybrid Zone

We designed the hybrid zone experiment (HybZon) to isolate genetic and environmental effects on DNA methylation variation while maintaining age and sex constant. We sampled 3 purebred male chicks of each taxon (distinct from ComGar) in addition to 16 hybrid chicks along a transect across the German hybrid zone during field trips in May 2008, 2013, and 2014. Hybrid individuals were characterised in a previous study according to genotype information of 1111 single nucleotide polymorphism (SNP) markers spread across the genome that include two of the major loci coding for plumage colour variation, as outlined in Knief et al. (2019) including a genetic factor on chromosome 18 (chr18) and the gene NDP on chromosome 1. Samples were chosen to represent the full variation of genome‐wide admixture (range of hybrid index: 0.0—1.0). With the inclusion of the purebred samples, hybrid indices thus both covered the full range from 0.0 (*C*. (*c*.) *corone*) to 1.0 (*C*. (*c*.) *cornix*) as represented by blood samples from 22 male individuals with an age range of 7–25 days (Table S1). Hatchlings were sampled from different nests to ensure unrelated individuals were sampled, and extra pair paternity in crows is low (Knief et al. 2020).

### 2.4 Methylomic Sequence Data

We assessed genome‐wide 5mC DNA methylation in whole blood with reduced‐representation bisulfite sequencing (RRBS) involving two separate sequencing efforts for the ComGar and HybZon experiments. For the ComGar, genomic DNA was isolated using QuickExtract kits (Epicentre, Illumina) for library preparation (300 ng; *n* = 24 representing three age classes of each sex and taxon at parity). Libraries were created by adapter‐ligating and end‐repairing *MspI* digested fragments following the NEBNextUltra protocol (New England BioLabs). Bisulfite conversion was performed with the EZ DNA Methylation Gold Kit (Zymo) and bisulfite‐converted DNA was then amplified with the NEBNext Universal primers and NEBNext index primers using 12 PCR cycles. Libraries were cleaned twice using AMPureXP beads (55 μL beads to 50 μL sample). A 0.5% spike of non‐methylated lambda phage DNA was included in the libraries to confirm bisulfite conversion efficiency (Lea et al. 2016). Final libraries were evaluated using a TapeStation with the HS D1000 kit. Adapter‐ligated fragments were quantified with qPCR using a library quantification kit for Illumina (KAPA Biosystems/Roche) on a CFX384Touch instrument (BioRad) prior to cluster generation and sequencing. RRBS libraries were sequenced on a NovaSeq 6000 using an SP Flowcell, single‐end 100‐*bp* read lengths using v1 sequencing chemistry, including a 25% PhiX spike‐in. Sequencing was performed by the SNP&SEQ Technology Platform in Uppsala, which is part of the National Genomics Infrastructure (NGI) Sweden and Science for Life Laboratory. In addition to this sequencing effort, we generated 22 RRBS libraries from the HybZon experiment at a separate facility (Novogene, Co. Ltd.). RRBS *MspI*‐fragmented libraries were created similarly to the common garden experiment detailed above, except the final libraries were sequenced paired‐end on a NovaSeq 6000 with 150‐*bp* read lengths.

### 2.5 Genome Reference and Genomic Feature Annotation

All analyses were performed on the chromosome‐level hooded crow (*C*. (*c*.) *cornix*) reference genome (National Centre for Biotechnology Information accession number: ASM73873v5) (Weissensteiner et al. 2020). Analyses were carried out on all named chromosomes (i.e., excluding unplaced scaffolds). Genomic features were extracted as follows. We annotated the reference genome into non‐overlapping tracks of promoter regions, repeats, coding sequence (CDS), intronic, and intergenic sequence (Figure S1). Promoter regions were identified as CpG islands located within 2‐*Kb* of a gene's start coordinates. While this strategy will likely fail to identify many true promoters, particularly those with promoters not directly within the proximity of the transcription start site (TSS) (Ioshikhes and Zhang 2000), it will provide a conservative estimate of CpG islands with direct relevance to local transcripts. We created a *de novo* CpG island track using makeCGI v1.3.4 (Wu et al. 2010), requiring a length > 200‐*Bp*, a GC content > 50%, and an observed/expected CG ratio > 60%, providing 30,459 islands. Only gene elements from the RefSeq.*gff* annotation file that intersected our CpG island track with the strand‐aware 2‐*Kb* region upstream of the gene start were retained, using bedtools v2.29.2 (Quinlan and Hall 2010). This resulted in 11,472 CpG‐promoter islands, from a total of 17,944 genes. We identified repetitive regions with RepeatMasker v4.1.1 (Smit et al. 2013) using the repeat library from chicken and excluding simple repeats (‘‐nolow’). Genic CDS and introns were extracted directly from the RefSeq annotation. A non‐overlapping annotation feature track was created with bedtools and R v4.1.1 (R Core Team 2017) by prioritising promoter regions, repeats, CDS, introns, and intergenic annotations, in that order (Figure S1).

### 2.6 Quantification of Genetic Variation

Genetic polymorphisms involving C‐T and A‐G transitions are problematic for bisulfite sequencing experiments because C‐T and A‐G mismatches between sequence and reference are used to identify DNA methylation (Barrow and Byun 2014; Krueger and Andrews 2011). We therefore exploited an existing population resequencing dataset from 28 male hooded and carrion crows sourced from the same allopatric populations (Uppsala, Sweden and Konstanz, Germany) analysed in this study (*n =* 14 of each taxon) to identify transition SNPs. While these individuals are not directly represented within the bisulfite sequencing datasets, previous research has supported the stability of population genetic structure within these geographic regions (Knief et al. 2019; Metzler et al. 2021; Poelstra et al. 2014; Vijay et al. 2016). We also used this reanalyzed resequencing dataset to quantify genome‐wide genetic variation (*F*_ST_, *D*_XY_, Tajima's *D*, haplotype diversity, Fu & Li's D*) to assess its relationship with methylation variation. In short, 130 paired‐end Illumina libraries for the 28 samples were adapter‐trimmed with BBTools v38.18 (Bushnell 2021), aligned to the reference with BWA v0.7.17 (Li and Durbin 2009), merged with samtools v1.7 (Li et al. 2009), and deduplicated with GATK v4.1.9.0 (Auwera et al. 2013). After read trimming and filtering, alignment to the same genome as above resulted in a genome‐wide mean coverage of 15.1× (range 8.03–30.9×), calculated in 100‐*Kb* windows with mosdepth v0.3.1 (Pedersen and Quinlan 2018) (Table S2).

We created an all‐sites variant file with bcftools v1.16 (Danecek et al. 2021). For filtering variant sites, we retained only biallelic sites passing hard filter thresholds (‘QUAL > 20, DP < 2*Average DP, or DP > 28×’), which had scored genotypes in at least 90% of individuals (*n =* 25/28 with *FMT/DP* ≥ 3). Following hard site‐level and genotype‐level filters, we retained 9.44 million variant sites and 1.01 billion invariant sites. Coordinates for transitions (C‐T and A‐G polymorphisms) were subset using bash scripts for methylation site masking. Population genetic metrics for *C*. (*c*.) *corone* and *C*. (*c*.) *cornix* crows (*F*_ST_ and *D*_XY_ between taxa and Tajima's *D*, haplotype diversity, and Fu & Li's D* among all individuals) were calculated in 5‐*Kb* windows (‘–windType sites–windSize 5000’), requiring a minimum of 1000 shared sites across samples (‘–minSites 1000’) using the genomics general repository (Martin et al. 2019) for *F*_ST_ and *D*_XY_, and the *Theta_D_H.Est* repository for the remaining metrics (Pan et al. 2022). Principal components analysis (PCA) on linkage disequilibrium (LD)‐pruned genome‐wide SNPs was performed with SNPRelate v1.28.0 (Zheng et al. 2012), and again exclusively on the SNPs falling within the *focal region* on chromosome 18 (Figure S2). Genetic data were visualised with karyoploteR v1.20.0 and ggplot2 v3.3.6 (Gel and Serra 2017; Wickham 2016).

### 2.7 Quantification of 5mC Methylation

All bisulfite sequence datasets were first trimmed with trim_galore v0.6.7 (Krueger 2012), ensuring the artificial filled‐in positions were trimmed in the RRBS datasets (‘–rrbs’), and requiring average read Phred scores of at least 20 (‘–quality 20’). Bisulfite conversion was successful, measured using a non‐methylated lambda phage DNA spike into each library (minimum, mean, maximum conversion rate: 98.90%, 99.27%, 99.74%, Table S3). Reads were aligned to the reference genome using Bismark v0.23.0 with default settings (Krueger and Andrews 2011). We extracted CpG methylation calls using Bismark, iteratively repeating this process after ignoring read positions exhibiting systematic methylation biases, evidenced through methylation bias plots (Figure S3). We removed any sites overlapping a C‐T or A‐G SNP (identified from the genetic resequencing experiment, see above) using bedtools v2.29.2 (Quinlan and Hall 2010) or located on a scaffold (as we only analysed sites on assigned chromosomes), and removed any sites covered by less than 10 reads in each individual across all experiments. Methylation levels (%) were consistent across read positions after trimming, indicating no systematic positional read biases, although high coverage spikes in our RRBS datasets lead us to set upper coverage limits, for which we removed a conservative degree to alleviate any concerns with repetitive fragments that would be difficult to map with A‐T rich bisulfite sequencing reads (remove the top 1% coverage outliers across all experiments). Finally, CpGs were removed from the experiment if they had missing data from more than 3 individuals in the ComGar (*n =* 21/24) or HybZon (*n* = 19/22) experiment.

### 2.8 Covariants of Methylome Variation

The primary aim of this study was to investigate how DNA methylation varies across taxa and to assess the extent to which this variation covaries with underlying genetic differentiation. While we accounted for potential confounding influences of physiological and environmental factors, our analytical focus centred on disentangling the relationship between genetic and epigenetic variation. To this end, we employed (i) multivariate analyses to assess global physiological and environmental contributions to methylation variation; (ii) beta‐binomial regressions to identify base‐pair resolution methylation differences associated with taxon and hybrid index, while controlling for physiological and environmental covariates; and (iii) supervised machine learning complemented with bootstrapped correlation analysis to quantify the covariation between genome‐wide genetic variation, genome architectural features, and DNA methylation patterns (Figure 1). Methylation data were treated according to the method requirement as a methylation proportion (ranging from 0.0% to 100% for distance‐based redundancy analyses and machine learning regressions), or directly as methylated and non‐methylated read counts (for DMP detection with beta‐binomial regressions). We ensured analytical insensitivity by repeating analyses, where applicable, using multiple methylation inputs (e.g., analysed the machine learning problem using both the mean methylation value and the maximum observed value in 5‐Kb windows, and approached it as a classification problem instead of a regression using a binary response, e.g., methylated or non‐methylated).

#### 2.8.1 Multivariate Analyses

To assess the effects of age, sex, and taxon on DNA methylation patterns we used two approaches: multivariate statistics examining global correlations of methylation variation (distance‐based redundancy analyses; dbRDA), and differentially methylated position (DMP) analyses allowing for ultra‐fine resolution of single differentially methylated CpGs. First, for each experiment independently (ComGar, HybZon), we examined global DNA methylation variation with a dbRDA constrained ordination. Methylation input for the ordination was a matrix composed of DNA methylation proportions with columns corresponding to each individual CpG site and rows corresponding to individual samples. Euclidean distance as the optimal distance measure for dbRDA for each experiment was identified using Spearman's rank correlations within vegan v2.5‐7 using the rankindex function. A pre‐processing data script (i.e., recipe) was created with tidyverse v1.3.1 and tidymodels v0.1.4 (Kuhn and Wickham 2020; Wickham et al. 2019), ensuring variables were in proper classes. Scaled dbRDAs were then implemented with vegan, and the contributory effects of each variable were assessed with an ANOVA by term with 10,000 permutations (Table S4). Adjusted pseudo‐*R* ^2^ and overall model significance as assessed with an ANOVA are reported.

#### 2.8.2 Beta‐Binomial Regression

We then determined the physiological and environmental determinants of DNA methylation at CpG‐resolution using DMP detection directly from methylated and non‐methylated read counts. Explanatory variable effect estimates (test‐statistics) and FDR‐corrected *p*‐values were obtained for each variable and for each experiment using a beta‐binomial regression with an arcsine link implemented in the R package DSS v2.42.0 (Park and Wu 2016). Explanatory variables for the ComGar were age (Chick, Yearling, Adult), sex (Male, Female), and taxon (*C*. (*c*.) *corone* and *C*. (*c*.) *cornix*); for HybZon were genetic ancestry hybrid index and sampling year (Table S1).

Next, we validated DMPs across experiments. Within each experiment, individual CpG sites were assigned a corresponding classification (e.g., age, sex, taxon), or were marked as invariable (less than 10% variation observed across the entire experiment), or indeterminate (no significant variation corresponding to any of the covariates). Classifications were based on an FDR detection threshold below 1%, non‐significance for other covariates (FDR > 1%), and a conservative minimum divergence between group methylation means of variables of interest of 25% (e.g., absolute value of mean_Male5mC_—mean_Female5mC_ > 25%). Concordance between FDR‐corrected *p*‐values and methylation divergence for each effect was checked with volcano plots (Figure S5). No absolute divergence thresholds were used for continuous covariates (e.g., hybrid index) as these were not factor variables and did not contain groups.

**FIGURE 3:**
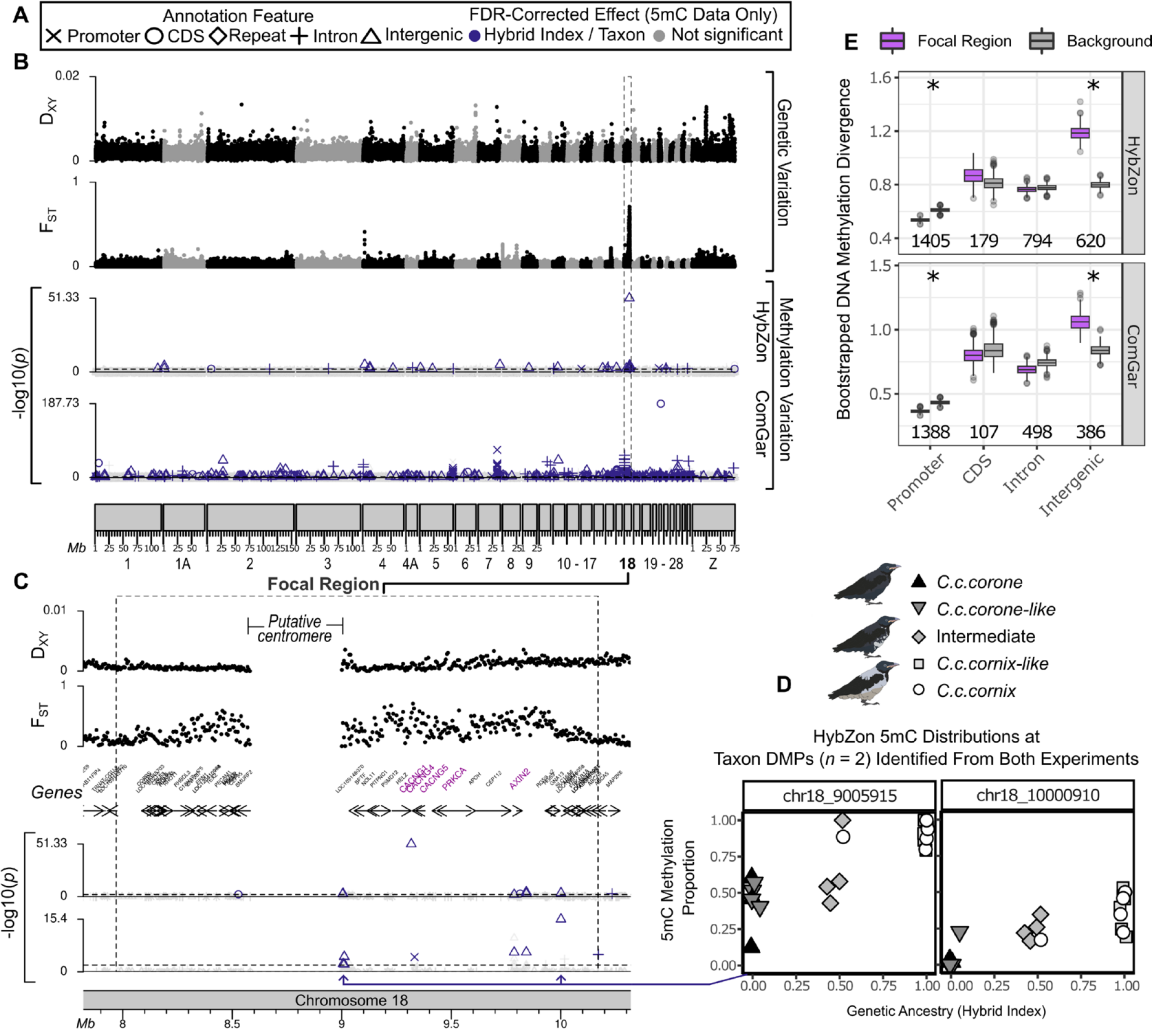
Methylomic variation along the crow genome. (A) Legend for genomic annotation features (symbols) and explanatory variables (colours) for each differentially methylated position (DMP). (B) Genetic variation: Genetic divergence (*D*_XY_: Min 0, mean 0.0015, max 0.013) and differentiation (*F*_ST_: Min 0, max 0.71) across 28 allopatric hooded (*Corvus* (*corone*) *cornix*) and carrion crows (*C*. (*c*.) *corone*) reanalyzed from whole genome resequencing data. Chromosome 18's elevated *F*_ST_ region (*focal region*) is highlighted, with 99% of sites having *F*_ST_ > 0.3. Methylation variation: FDR‐corrected *p*‐values indicate taxon‐specific DMPs (*p* < 0.01) after covariate control (cf. Figure 2). (C) *Focal region* with high genetic differentiation (*F*_ST_ = 0.298 vs. 0.010) contains the only two taxon‐related DMPs identified in both ComGar and HybZon experiments, with candidate genes for plumage polymorphism (Knief et al. 2019) in larger purple text. (D) Methylation levels (*y*‐axis) track genetic ancestry (*x*‐axis) at two taxonomically‐related sites, showing hypomethylation in carrion crow ancestry (Figure S11). (E) Methylation divergence of CpGs in the *focal region* compared to autosomal background using bootstrap sampling. Boxplots show first and third quantiles, with significance if 95% distribution tails don't overlap. Total CpGs in the *focal region* indicated below boxplots.

Classified CpGs were then merged across experiments at base‐pair resolution. As our primary focus is on the association between taxonomic DNA methylation and genetic variation, we focused on DMPs which were identified as taxonomically related in both the hybrid zone (HybZon) and common garden (ComGar) experiments. We identified two CpGs which were significantly associated with taxon across both experiments (Figure 3B,C and Figure S6), all within the *focal region* on chromosome 18. While this approach relies on CpG overlap between experiments, which is of course limited by coverage and technical conditions, we preferred to highlight these inter‐experimental taxon DMPs as they serve as a repeatability metric within a single study. Furthermore, as our primary interest is on DNA methylation divergence between taxa, integrating two metrics of taxonomic divergence (i.e., parental comparisons in two experiments and hybrid index in another) will identify sites most likely corresponding to taxonomic divergence with relevance to speciation in this system (Knief et al. 2019). While financial constraints may cause our study to miss small‐effect DNA methylation loci due to sample size limitations, our sample size within each experiment matches those of other RRBS studies (Capra et al. 2023; El Kamouh et al. 2024; Lundregan et al. 2022; Metzger and Schulte 2017; Meyer et al. 2023). Our inter‐experimentally validated approach thus offers a novel framework to assess both inter‐experimental batch effects and repeatability in a conservative framework.

Lastly, we assessed taxonomic differences in DNA methylation within the *focal region* compared to the autosomal background using bootstrapped sampling with replacement within R (R Core Team 2017). Specifically, for each DNA methylation experiment and each genomic annotation feature (e.g., promoter, intergenic), we sampled a number of autosomal CpGs equal to the number of CpGs within the *focal region* for that data subset and calculated the mean taxon effect estimate (i.e., test statistic from beta‐binomial regressions), repeating this process 1000 times. We considered the comparisons between the *focal region* and the genomic background to be significant if the 95% confidence intervals did not overlap. To control for the effect of mutations altering the CpG context of neighbouring cytosines on either strand, we also repeated this analysis after excluding all methylation calls adjacent (1*‐Bp* upstream and 1*‐Bp* downstream) to any known SNP, including both transitions and transversions, as identified in the whole‐genome resequencing dataset (Figure S7).

We then assessed the chromosomal distribution of DMPs by plotting the count of DMPs by chromosome for both ComGar and HybZon experiments. Visual inspection suggested chromosome 18 had a higher‐than‐expected number of DMPs in both experiments, particularly in HybZon (Figure S4). To evaluate whether individual chromosomes, including chromosome 18, harboured a significantly elevated proportion of DMPs, we performed binomial tests comparing observed versus expected DMP proportions for each chromosome in each experiment. For each experiment, we calculated the total number of taxonomically associated DMPs across all chromosomes and the number observed on each specific chromosome. The expected proportion of DMPs per chromosome was computed as the number of assayed CpGs per chromosome divided by the total CpGs in each experiment, assuming DMPs are randomly distributed in proportion to genomic content. For each chromosome–experiment combination, a binomial test was conducted using the number of observed DMPs on that chromosome as the number of successes, the total number of DMPs in that experiment as the number of trials, and the expected proportion based on assayed CpGs as the probability of success. We extracted the observed proportion of DMPs and associated 95% confidence intervals and identified chromosomes with lower confidence limits exceeding the expected proportion as significantly enriched for taxon DMPs. We also repeated the above analysis except focusing specifically on sex‐associated DMPs, using a binomial test to evaluate whether the Z chromosome harboured a significantly different number of sex‐related DMPs than expected based on assayed CpGs on the Z chromosome compared to the total assayed CpGs in the ComGar.

#### 2.8.3 Supervised Machine Learning and Bootstrapped Correlation Analysis

We sought to quantify the relationship between taxonomic DNA methylation divergence and its chromosomal substrate at genome‐scale, namely using genome properties and population genetic variation. We performed this analysis because DNA methylation data is scarcely analysed in the context of even a single population genetic estimator, and to see if DNA methylation was predictable with these features alone. We averaged measures of population genetic variation (*F*_ST_, *D*_XY_, Tajima's *D*, haplotype diversity, Fu & Li's D*; see above *Quantification of Genetic Variation*) in non‐overlapping genomic windows of 5‐*Kb* (Figure S8). In addition, we collated information on genome properties such as genomic annotation, GC‐content, relative position of the window along a chromosome, and chromosome length. As measure methylation divergence we used the parameter estimates from the beta‐binomial models (see above *Beta‐binomial Regression*). For the ComGar experiment this corresponds to the parameter estimate of the taxon effect controlling for sex and age, for the HybZon experiment it corresponds to the effect of hybrid index controlling for year. Methylation divergence was calculated for each CpG site and then averaged within non‐overlapping 5‐*Kb* windows spread across the genome. For sensitivity, we also repeated the analyses with maximum divergence values within 5‐*Kb* windows instead of the mean and arrived at similar conclusions, except that the importance for *promoter* regions was relinquished into GC‐content, highlighting the interconnected nature of these covariates (Figure S9). We imputed population genetic values from the nearest 5‐*Kb* windows where values did not exist (maximum number of missing 5‐*Kb* windows across experiments: *n* = 356, ≤ 1.0%), seemingly more common in areas of high GC‐content (within missing windows = 68.2%, outside = 60.7%; Table S5). We then assessed the raw distributions of our DNA methylation divergence response variable within each experiment and deemed a log transformation appropriate (Figure S9). Collinearity between explanatory variables was low across both experiments (minimum, mean, maximum: −0.21, 0.056, 0.62; Table S6).

We assessed the relationship between DNA methylation divergence, genome properties, and population genetic variation with supervised machine learning using random forests with ranger v0.14.1 (M. N. Wright and Ziegler 2017) and boosted regression trees with XGBoost v1.7.1.1 (Chen and Guestrin 2016). Pre‐processing and modelling used a tidyverse v1.3.1 and tidymodels v0.1.4 (Kuhn and Wickham 2020; Wickham et al. 2019) framework implemented within R v4.1.1 (R Core Team 2017) using normalised numerical covariates. Datasets were then stratified into 75% training and 25% testing partitions to be analysed with 5‐fold cross validation. Model parameters (‘trees, min_n, mtry’; and ‘learn rate’ for XGBoost) were tuned to maximise *R* ^2^ with finetune v1.0.1 and caret v6.0‐93 (Kuhn 2008; Kuhn and Wickham 2020) using a random grid search. Workflows were finalised and implemented with tidymodels using the tuned parameters, allowing for final extraction of covariate permutation importance using vip v0.3.2 (Greenwell and Boehmke 2020) and model fit (root mean squared error, RMSE) (Table S7). Permutation importance measures explanatory variable importance by shuffling each covariate's values and measuring the impact on model fit, and is a better gauge of contributory effects than alternative methods relying on impurity, a metric of covariate importance which measures importance based on how well the variable splits the data in the model (Altmann et al. 2010). Permutation importance is therefore a useful measure to determine the importance of each covariate, but only insofar as the final models have sufficient overall explanatory power. To generate confidence intervals on covariate importance and model explanatory power, we replicated the entire process for all DNA methylation experiments using different random seeds. We then scaled permutation importance within each iteration to a value of 1.0 to allow for comparisons across the two regression engines. Furthermore, to see if the problem could be approached more parsimoniously with supervised classification techniques, we transformed our continuous DNA methylation divergence response variable into a discrete binary response and repeated the entire process again (Data S1 and Figure S9), this time assessing models with accuracy and covariate permutation importance, and observed the same patterns (Figure S9 and Table S7).

**FIGURE 4:**
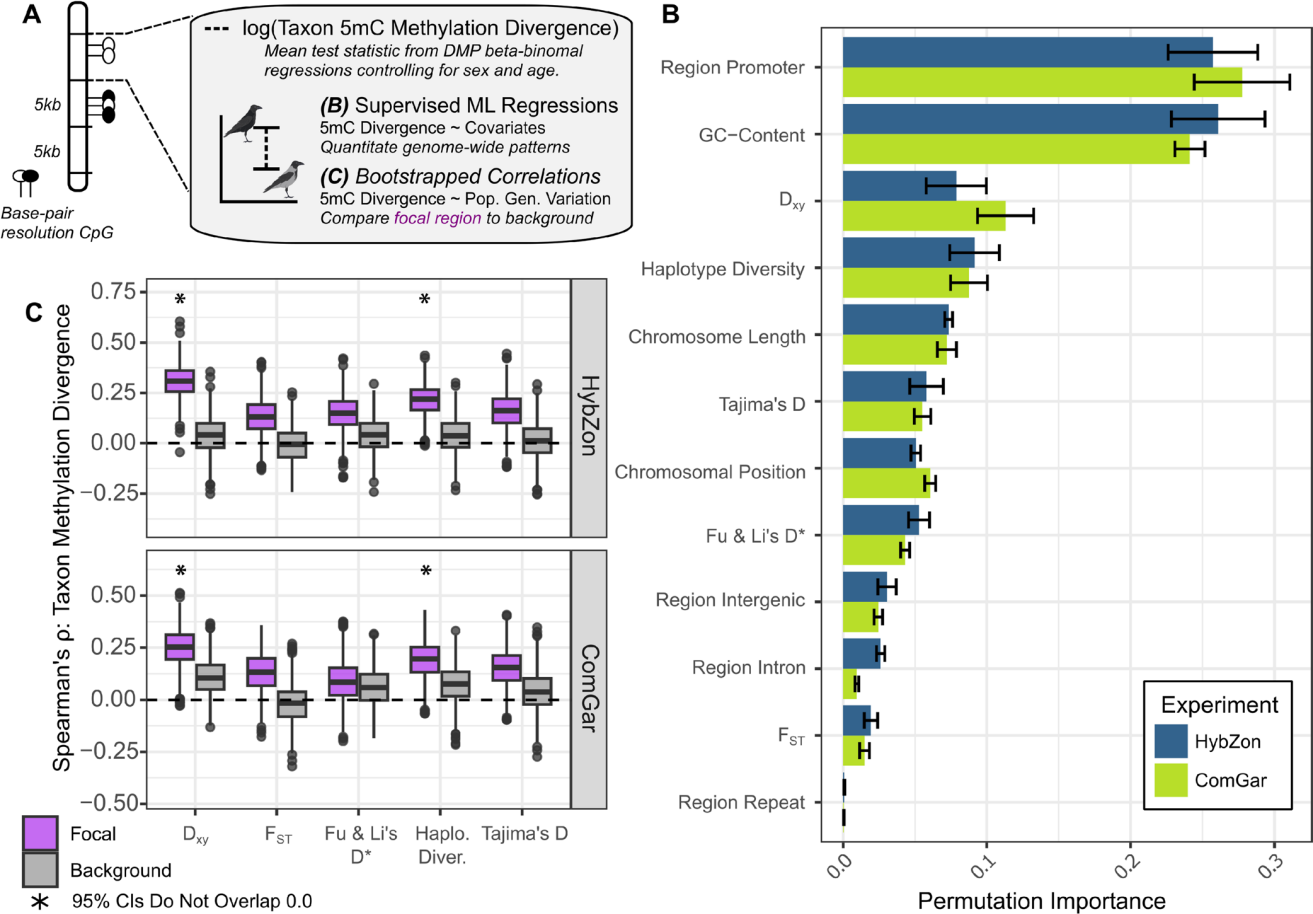
Predicting 5mC methylation divergence between hooded and carrion crows. (A) Schematic depicting the two methods used to examine broad chromosomal and population genetic patterns associated with DNA methylation. (B) A supervised machine learning regression approach across the two methylation sequencing efforts determined the relative contributions of explanatory variables (*y*‐axis) to taxonomic methylation divergence. Methylation divergence was summarised as the binned mean of parameter estimates for taxon effects in the beta‐binomial models controlling for environmental factors or sex and age in the HybZon and ComGar experiment, respectively (cf. Figure 3). Permutation importance measures variable importance against a decrease in model fit and indicates predictive ability. (C) Spearman's rank correlation between DNA methylation divergence and population genetic metrics within and outside of the *focal region* on chromosome 18, indicating elevated covariation within the *focal region* which is characterised by a higher degree of genetic differentiation. Distributions drawn from 1000 bootstrap sampling events; default parameter boxplots correspond to first and third quantiles; significance indicated if either side of the 95% distribution tails do not overlap zero.

We complemented our supervised machine learning approach with a bootstrapped Spearman's rank correlation analysis between taxonomic DNA methylation divergence and population genetic variation, with a focus on how patterns differ between the *focal region* on chromosome 18 and the autosomal background. The boundaries of the *focal region* were established by the start and end of windows on chromosome 18 with elevated genetic differentiation (*F*_ST_ > 0.3). At this *F*_ST_ threshold nearly all 5‐*Kb* windows were localised on chromosome 18 (99.0%, *n =* 149), and the resulting region (starting at 8.07e6 and ending at 10.07e6; 2‐*Mb*) corresponds to the length of the island identified previously, although on a different genome assembly (Knief et al. 2019; Poelstra et al. 2015). For each DNA methylation experiment, we sampled (with replacement) autosomal windows equal to the number of focal windows and calculated Spearman's rank correlation between DNA methylation divergence (absolute value of test‐statistic estimates for taxon effects from DMP analyses) and population genetic measures inside and outside of the focal region. We considered the correlations to be significant if their 95% quantile distributions did not overlap zero (Figure 4C).

## 3 Results and Discussion

### 3.1 Ontogenetic Program

We first assessed global variation in DNA methylation patterns, focusing on taxonomic divergence, and subsequently examined physiological and environmental contributions. In the ComGar experiment (see Figure 1), age class, sex, and taxon collectively explained a moderate amount of methylation variation (adj. *R* ^2^ = 0.13, all *p* < 0.01), with the most pronounced contrast between chicks (mean age = 22.1 days) and mature individuals (mean age = 922.9 days). Among the predictors, all but the yearling age class were significantly associated with methylation variation (all *p* = 0.001, except *p* = 0.987 for yearlings; Figure 2A). Notably, the inclusion of taxon increased model fit substantially (adj. *R* ^2^ = 0.136 vs. 0.093 without taxon), highlighting a contribution of taxon differences to overall methylation divergence (*F* = 2.01, *p* = 0.001), even after accounting for ontogenetic and sex‐specific effects. In the HybZon experiment (see Figure 1), taxon differences—quantified via a continuous hybrid index reflecting genetic ancestry—were also significantly associated with overall methylation variation (*F* = 1.19, *p* = 0.038), albeit explaining a modest proportion of the total variance when combined with sampling year (adj. *R* ^2^ = 0.032 vs. 0.027 without hybrid index; Figure 2B).

At the CpG level, differentially methylated positions (DMPs) recapitulated the global patterns in the ComGar experiment: the majority were associated with age (*n* = 7250), followed by sex (*n* = 2768), and taxon (*n* = 639). This rank order suggests that, although age‐related effects are dominant within the experimental context, taxonomic divergence is discernible after accounting for other ontogenetic effects. To further validate these results, we analysed an independent whole‐genome bisulfite sequencing (WGBS) dataset derived from the ComGar experiment, encompassing variation across tissue types. Consistent patterns emerged, with tissue identity exerting a strong influence on methylation variation (dbRDA adj. *R* ^2^ = 0.365; *n* = 148,364 tissue DMPs; Data S1 and Figure S10), in line with established roles for DNA methylation in cell fate determination (Gama‐Sosa et al. 1983; Izzo et al. 2020). In addition to reinforcing the contributions of methylation to developmental processes and sex‐specific regulation (de Paoli‐Iseppi et al. 2019), our data revealed that 163 of sex‐associated DMPs were located on the Z chromosome, significantly more than would be expected based on assayed CpGs (binomial test ComGar: DMP_exp_ = 0.027, lower 95% CI DMP_obs_ = 0.050, *p*‐value = 5.74e‐20). While DNA methylation appears largely unrelated to mechanisms of partial dosage compensation in crows (Catalán et al. 2023), this clustering could suggest a role for DNA methylation in mediating gene‐specific sex bias in gene expression.

### 3.2 Methylomic and Genomic Interplay

Methylation divergence separating the diverging taxa was observed across both experiments. In the ComGar and HybZon experiments, we identified 596 and 45 taxon‐related DMPs, respectively (Figure 3B,C). To reduce the number of potential false positives common to differential methylation analyses (Mueller et al. 2025), we focused on DMPs that were present in both experiments. Two taxon‐related DMPs survived this dual experimental validation. They were both intergenic, fell into the *focal region* of elevated genetic differentiation (Figure 3C) and showed hypomethylation of carrion crow ancestry (Figure 3D and Figure S11). Encoding of methylation genotypes with k‐means clustering at these loci identified strong methylation linkage disequilibrium between these two sites (*D* = 0.136, *D*′ = 0.999) (Figure S12). Note, however, that these two DMPs, while not intersecting SNPs from the whole genome resequencing dataset exactly, do occur in adjacent positions to known SNP variants that are near fixed between carrion and hooded crows. Methylation divergence and linkage disequilibrium thus reflect the direct effect of genetic variation altering the CpG context of the mutated haplotype.

More generally, chromosome 18 was the only chromosome to harbour significantly more DMPs than expected under a binomial null model in both experiments, with observed counts exceeding the 95% confidence interval of the expected distribution (ComGar: DMP_exp_ = 0.027, lower 95% CI DMP_obs_ = 0.042, *p*‐value = 1.56e‐08; HybZon: DMP_exp_ = 0.029, lower 95% CI DMP_obs_ = 0.170, *p*‐value = 5.69e‐12) (Figure S4). This effect was not strongly influenced by the direct effect of CpG context altering SNPs. Methylation positions adjacent to known SNPs were overall rare (focal region: *n =* 5 out of 2933 HybZon; *n =* 3 out of 2376 ComGar; background: 10,270 out of 1,363,877 HybZon; *n* = 4296 out of 1,021,810 ComGar) and their exclusion did not change the result (Figure S7). Elevated methylation divergence in the *focal region* is thus robust to local genetic variation at neighbouring CpG sites, revealing an association between *cis*‐acting genetic variation and DNA methylation beyond the most obvious mutations altering the CpG context. Still, these findings highlight the need to integrate DNA methylation data with whole‐genome resequencing to account for the influence of SNPs, which inherently reflect underlying patterns of genetic structure (Mueller et al. 2025).

The fact that these taxonomically relevant DMPs cluster in proximity to the plumage polymorphism candidates AXIN2, PRKCA, and an array of CACNG genes (Knief et al. 2019) supports the notion that phenotypically relevant genetic variation and DNA methylation may indeed covary as a result of divergent selection, as has been proposed in white‐throated sparrows (Sun et al. 2021) (but see Heckwolf et al. 2020). DNA methylation may thereby act as a mediator translating signals of *cis‐*genetic variation into differential physiological activity. Note, however, that the taxonomic DMP loci were identified using blood tissue which is not histologically relevant to melanin production in crows (C.‐C. Wu et al. 2019), and would therefore reflect a tissue‐unspecific, pleiotropic signal. Moreover, none of the DMPs overlap any genes or promoters directly, and more generally, elevated methylation divergence was restricted to intergenic space while it was decreased in promoters compared to the autosomal background (*p* < 0.05 in both experiments; Figure 3E and Figure S10D). Altogether, this renders the functional importance of the 5mC DMPs in the *focal region* less likely and instead points to a mere association with *cis‐*genetic variation.

Having established covariation of local genetic divergence on 5mC methylation, we next assessed whether taxonomic variation in DNA methylation could be predicted more broadly. Using supervised machine learning regressors, we tested whether DNA methylation divergence between taxa could be reliably predicted with chromosomal features (e.g., GC‐content, chromosomal positioning) or population genetic variation (e.g., *F*_ST_, haplotype diversity) (Figure 4A). Methylation divergence was quantified as the mean test‐statistic for taxon effects from the beta‐binomial model within 5‐*Kb* genomic windows, matching the resolution of the population genetic data (see Material and Methods). Methylation divergence was predicted with moderate to good prediction accuracy (RMSE 0.17–0.24, Table S7) and was in part predicted by genome properties (chromosome length, positioning along chromosome, functional annotation) and to a lesser extent by measures of population genetic variation (*F*_ST_, *D*_XY_, haplotype diversity, Tajima's *D*, Fu & Li's D*) (Figure 4B). Overall, GC‐content (Permutation Importance 95% CI intervals across all experiments: 21.0%–29.0%) and promoter regions (22.0%–32.0%) were the strongest predictors indicating decreased methylation divergence within GC‐rich CpG islands near putative promoter regions (Figure 4C). This finding corroborates the prevalent conservation of evolutionary processes at CpG islands with a putative functional role in gene regulation in vertebrates (Bird et al. 1985; Long et al. 2016). Relative positioning along a chromosome (4.90%–6.20%) and chromosome length (6.50%–8.00%) were weaker predictors of methylation divergence, yet lend support for methylome divergence proceeding more rapidly on micro‐chromosomes or near chromosome ends where recombination is substantially higher (Kawakami et al. 2014; Weissensteiner et al. 2017). Further supporting the relationship between genetic variation and DNA methylation, permutation importance was highest for a measure of genetic divergence *D*_XY_ (6.40%–13.0%), followed by haplotype diversity (6.70%–11.0%) and a measure of the allele frequency spectrum Tajima's *D* (4.30%–7.00%), and lastly, genetic differentiation *F*_ST_ (1.10%–2.30%) (Figure 4B).

These results indicate an interplay between population genetic variation and DNA methylation and support previous research identifying population‐level correlates between genetic and DNA methylation variation (Carja et al. 2017; Wang et al. 2022), particularly in regions undergoing selection (Shirai et al. 2021). To compare the relative strength and obtain directionality of the interplay between DNA methylation and population genetic variation, we examined bootstrapped correlations within the *focal region* compared to the autosomal background. As expected, the relationship between DNA methylation and genetic variation was stronger within the *focal region*, particularly for *D*_XY_ and haplotype diversity (Figure 4C). Consistent with these analyses, results from ancillary WGBS data using additional common garden crows corroborated strong effects of GC‐content and *D*_XY_ on taxonomic DNA methylation divergence (Data S1; Figures S10D and S13).

In summary, genome properties (e.g., GC‐content) and segregating genetic variation both contribute to taxonomic divergence of DNA methylation, most pronounced in the *focal region* undergoing divergent selection. These results are overall consistent with a general carry‐over effect of *cis‐*acting genetic variation on patterns of methylation variation, as observed within *Papio* baboons and other songbirds (Boman et al. 2024; Vilgalys et al. 2018). Hitchhiking 5mC variants in linkage disequilibrium with *cis‐*acting genetic variation may or may not be co‐opted functionally (Hawe et al. 2022; Min et al. 2021; Taudt et al. 2016; Yagound et al. 2019) (for an example of *trans‐*acting genetic variation see (Höglund et al. 2020)). Conversely, linkage disequilibrium could be reinforced by selection on DNA methylation, dragging along genetic variation. The known causal effects of DNA methylation on genetic variation are currently limited to increased deamination of methylated cytosines—a process with potential evolutionary implications (Cooper and Krawczak 1989; Feinberg and Irizarry 2010; Hanson et al. 2022)—and this effect is particularly pronounced within transposable elements (Zhou et al. 2020). However, in our system, there is little evidence for taxon‐specific TE divergence (Warmuth et al. 2022), and taxon‐associated intergenic methylation changes are primarily confined to a single large‐effect region known to harbour genetic variants underlying phenotypic divergence (Knief et al. 2019). Our results instead suggest that methylation divergence is more parsimoniously interpreted as a by‐product of genetic divergence, rather than a driver of functional differentiation.

## 4 Conclusion

Overall, this study illustrates the utility and limitations of methylomic approaches and advocates a multiple experimental framework for addressing complexities that underlie genetic‐epigenetic interactions in natural populations. While our experimental design precluded direct evaluation of the prevalence or heritability of spontaneous epimutations, it provides evidence that natural variation in DNA methylation is firmly associated with general genome features and physiological processes orchestrating aging and cell fate. The latter conforms to the original definition of epigenetics as ‘the interaction of genes and their products […] which bring the phenotype into being’ (Waddington 2008, p. 242). Taxonomically‐relevant DNA methylation under controlled conditions corresponded to chromosomal features and segregating *cis*‐acting genetic variation between species, tempering expectations for an independent role of epigenetic variation during nascent species divergence (Wang et al. 2022). Nonetheless, a significant association between DNA methylation patterning and sampling years within the hybrid zone experiment suggests the possibility of environmental effects impacting the epigenome, although with unclear intra‐ (soma‐to‐soma) and transgenerational (soma‐to‐germline) heritability or functional relevance. We conclude that the main source of methylation variation with relevance to speciation in natural populations of European crow is predetermined by chromosomal organisation and genetic variation and provides little scope for independent evolution at this early stage of species diversification. Our findings raise questions about the purported importance of autonomous epigenetic divergence in speciation, although claims of its importance are often made in systems where intragenerational phenotypic plasticity could plausibly be facilitated by epigenetic divergence (Venney et al. 2024). This conundrum highlights that broad claims regarding the significance of epigenetics during speciation lack meaning unless contextualised with environmental, genetic, and temporal factors, and underscores the importance of exploiting both controlled conditions and experiments in the wild (Mueller et al. 2025). Overall, the extent to which autonomous epigenetic divergence fuels speciation hinges on the degree of residual epigenetic variation surviving deterministic ontogenetic reprogramming (Morgan et al. 1999; Reik et al. 2001), which motivates further study in wild systems.

## Author Contributions

Conceptualization: Justin Merondun and Jochen B.W. Wolf. Methodology: Justin Merondun and Jochen B.W. Wolf. Investigation: Justin Merondun and Jochen B.W. Wolf. Visualisation: Justin Merondun and Jochen B.W. Wolf. Funding acquisition: Jochen B.W. Wolf. Project administration: Jochen B.W. Wolf. Supervision: Jochen B.W. Wolf. Writing – original draft: Justin Merondun. Writing – review and editing: Justin Merondun and Jochen B.W. Wolf.

## Ethics Statement

All applicable international, national, and/or institutional guidelines for the care and use of animals were followed. Permission for sampling of wild carrion crows was granted by *Regierungspräsidium Freiburg* (Aktenzeichen 55‐ 8852.15/05, 8852.15), Landratsamt Zwickau (364.622‐N‐Her‐1/14), Landratsamt Mittelsachsen (55,410,704 Beringungserl‐Voigt_14), Landratsamt Vogtlandkreis (364.622‐2‐2‐88841/2014), Landratsamt Meißen (672/364.621‐Kennzeichnung von Tieren 18935/2013), Landratsamt Bautzen (67.3‐364.622:13‐01‐Krähen), Landesdirektion Sachsen (24‐9168.00/2013‐4), Landesamt für Verbraucherschutz, Landwirtschaft und Flurneuordnung Brandenburg (23‐2347‐8a182008) in Germany and by Jordbruksverket (Dnr 30‐1326/10) in Sweden. Import into Sweden was registered with *Veterinäramt Konstanz* (Bescheinigungsnummer INTRA.DE.2014.0047502) and *Jordbruksverket* (Diarienummer 6.6.18‐3037/14). Animal husbandry and experimentation were authorised and inspected on site by *Jordbruksverket* (Diarienummer 5.2.18‐3065/13, Diarienummer 27–14) and ethically approved by the European Research Council (ERCStG‐336536). Crows were hand‐raised and thereafter tightly monitored by experienced staff including one animal caretaker present throughout the entire period of 2 years. The social behaviour of the group was monitored on several occasions daily, from close by during feeding and from outside the aviary. Health status was monitored at regular intervals by staff and external veterinaries. While aggressive behaviours over food access (e.g., threatening) or perching sites (e.g., displacement) could be observed, none of the crows showed aberrant behaviour or a visible indication of stress.

## Conflicts of Interest

The authors declare no conflicts of interest.

## Supporting information


Data S1.

Data S2.

## Data Availability

All associated sequence data are uploaded to the NCBI SRA with run accessions indicated in Table S1 under Bioproject PRJNA594256. All bioinformatic code and input files to reproduce the figures and tables within the manuscript are available at this GitHub repository https://github.com/EvoBioWolf/2025_CORVID_methylation, with large input files stored at the manuscript's Zenodo repository: https://doi.org/10.5281/zenodo.15243953.
