# Supplementary material for: DNA Methylation Reflects Cis‐Genetic Differentiation Across the European Crow Hybrid Zone
Source: Mol Ecol. 2025 Jul 10;34(21):e70026. doi: 10.1111/mec.70026 (PMC12573725; doi:10.1111/mec.70026)
Supplement: Supplementary file 1 — Data S1. [file MEC-34-e70026-s002.docx]

Supporting Information for

**DNA methylation reflects cis-genetic differentiation across the European crow hybrid zone**

Justin Merondun, Jochen B. W. Wolf

Corresponding author: j.wolf@bio.lmu.de

**Supplementary Information Includes:**

Supplementary Text

Figs. S1 to S16

Table Captions S1 to S7

**Other Supplementary Materials include:**

Tables S1-S7 (Tables.xlsx)

External Repository with Bioinformatic code: https://zenodo.org/doi/10.5281/zenodo.14641719

# Supplementary Text

Corroboration with Whole Genome Bisulfite Sequencing (WGBS)

We analyzed additional whole-genome bisulfite sequencing (hereafter ComGar.WGBS) data for four fully mature individuals from the same common garden (two *C. (c.) cornix*, two *C. (c.) corone*, one male and one female per taxon), each sampled across three tissues (blood, liver, spleen). Of these 12 libraries, the four blood samples from mature crows were biological replicates from the RRBS dataset for technical validation. The additional eight WGBS libraries for two more tissues of those same individuals further allowed identifying the expected pervasive number of regions associated with tissue-specificity including regions outside of promoter-enriched RRBS datasets.

Libraries were generated for four blood, two spleen, and two liver samples in two sequencing efforts in order to obtain the 12 libraries, analyzed together with four previously released WGBS datasets for *C. (c.) cornix* spleen and liver (Catalán et al., 2023). In the first sequencing effort, WGBS libraries were created from genomic DNA isolated from whole blood using QuickExtract kits (Epicentre, Illumina; 150 ng input; *n* = 4). WGBS libraries were created following the TruSeq DNA Methylation kit (Illumina Inc., EGMK91324) according to the manufacturers’ protocol. As with RRBS libarries, bisulfite conversion was performed with the EZ DNA Methylation Gold Kit (Zymo) and bisulfite-converted DNA was then amplified with the NEBNext Universal primers and NEBNext index primers using 12 PCR-cycles. Libraries were cleaned twice using AMPureXP beads (55 ul beads to 50 ul sample). A 0.5% spike of non-methylated lambda phage DNA was included in WGBS libraries to confirm bisulfite-conversion efficiency and libraries were evaluated using a TapeStation with the HS D1000 kit. Adapter-ligated fragments were quantified with qPCR using a library quantification kit for Illumina (KAPA Biosystems/Roche) on a CFX384Touch instrument (BioRad) prior to cluster generation and sequencing and were sequenced paired-end on a HiSeqX with 150-Bp read length using v2.5 sequencing chemistry, including a 5% PhiX spike-in. Sequencing was performed by the SNP&SEQ Technology Platform in Uppsala which is part of the National Genomics Infrastructure (NGI) Sweden and Science for Life Laboratory.

In addition to this sequencing effort, we generated four supplemental WGBS libraries (an additional two *C. (c.) corone* tissues for each sex and supplemental sequencing for one individual from the previous WGBS effort) at a separate facility (Novogene, Co. Ltd.), bringing total sex, tissue, and taxon sampling to parity. The additional four WGBS libraries were generated at Novogene Co. Ltd. (Beijing, China), using the Accel-NGS® Methyl-Seq DNA library kit (Swift Biosciences) following the manufacturer’s instructions. These additional WGBS libraries were sequenced on a NovaSeq 6000 paired-end with 150-Bp read length. WGBS data was processed similarly to RRBS data, except post-bisulfite adapter tagged (PBAT) libraries required additional trimming before alignment to remove artificial bases inherent to the protocol (‘--clip_r1 9 --clip_r2 9 --three_prime_clip_R2 1’), and aligned reads were deduplicated with Bismark before CpG extraction (ComGar.WGBS mean and standard deviation CpG coverage: 15.9 ± 1.22x)

After filtering for CpGs supported by a minimum of 10 reads and less than 10% missingness across individuals within each experiment, we retained a total 1,089,024 ComGar.WGBS high-quality CpG sites. As expected, the WGBS approach showed a much lower proportion of invariable sites, where methylation variation exceeded 10% across all samples (9.8%). No batch effects associated with the two sequencing centers were apparent in the ComGar.WGBS experiment (Fig S14), identifying very repeatable measurements across sequencing centers (Spearman’s rho: 0.998 for all shared CpGs above 10x coverage). Replicated DNA extraction, library preparation, and sequencing for four biological replicates across both RRBS and WGBS protocols furthermore indicates repeatability among sequencing efforts (Fig S15). While correlations were lower between biological replicates across sequencing efforts than between technical replicate WGBS libraries (see above), these differences are likely explained by inherent differences between these protocols, sequencing coverage, and blood as a source tissue which may contain different cellular make-up in each extraction.

We repeated all analyses (*e.g.,* multivariate ordinations, DMP detection, machine learning predictions) on the ComGar.WGBS dataset. Minor analytical modifications included using Gower’s distance for dbRDA, instead of Euclidean, as evidenced by correlations (see methods). Tissue-specificity across blood, spleen, and liver dominated DNA methylation variation within this ComGar.WGBS experiment (dbRDA *p*-value for tissue effects; *p_(Tissue)_* < 0.01). Tissue explained a large proportion of overall methylation variation along with sex and taxon (overall dbRDA; *p* < 0.01, adj. R^2^ = 0.37), each having little effect by itself (*p_(Sex)_* = 0.10, *p_(Taxon)_* = 0.11) (Fig S10 and Table S4). Analyses repeated on the ComGar.WGBS dataset also indicated that taxonomic DNA methylation divergence was elevated within the *focal region—*also in intergenic space—and that *D_XY_*  and hapotype diversity are the most correlated with DNA methylation divergence within the *focal region* (Fig S10D and Fig S13).

Sensitivity of cut-site alignments in RRBS

Reduced representations protocols may suffer from off-target alignments which occur outside of a known digestive-enzyme cut-site. To reduce erroneous RRBS alignments, we performed a sensitivity analysis using a CpG methylation file which is intersected with all known *MspI* cut-sites on the reference genome. We generated this bed file with an *in silico* digest of the reference genome with *MspI* using SimRAD v0.96 (Lepais & Weir, 2014), requiring a fragment size between 40 – 350-Bp, providing 299,587 potential fragments with roughly 1.6M CG motifs, compared to the roughly 9.8M CG motifs in the entire reference. We then retained methylation calls on *MspI* fragments (for RRBS data only). RRBS reads were ensured to overlap an *in silico* digested *MspI* fragment (see above) using bedtools (Quinlan & Hall, 2010). Initial count inspections for RRBS data indicated a substantial increase in retained reads when overlapping *MspI* fragments at the alignment file (BAM) level (pre-methylation extraction), as opposed to *post-hoc* removal of individual sites after extraction, so extraction on RRBS dataset was repeated after filtering BAM files for *MspI* fragments, resulting in a total of 700,097 CpGs for the ComGar experiment and 833,936 CpGs for the HybZon experiment. All analyses were repeated to ensure corroborating results (Fig S16)

Relative methylation levels within focal region across hybrids

We further assessed DNA methylation variation among our hybrid individuals by examining distributions of 5mC methylation within the focal region compared to the genomic background for each hybrid group: unadmixed *C. (c.) cornix* and *C. (c.) corone*, hybrids which have hybrid indices closer to either parent, and hybrids intermediate to either. To compare methylation levels among hybrids and parentals within the *focal region* compared to the autosomal background, we performed permutation tests. We sampled an equal number of autosomal and focal region CpGs equal to the sites in the *focal region* with replacement and repeated this process 1,000 times, generating a distribution of the levels within each subset. Permutation tests between methylation levels within the *focal region* vs. the autosomal background corroborated the results found within Fig 3D, showing hypomethylation within carrion crows and their closely-related hybrids within the island of differentiation on chromosome 18, and hooded crows and their associated hybrids hypermethylated compared to the autosomal background (Fig S11). Similarly, hybrids with intermediate genetic ancestry coefficients exhibited intermediate methylation levels which did not differ from the autosomal background. No differences were observed between the focal region and the background within *Tissue* windows, serving as our negative control (Fig S11). This analysis was performed on the *MspI* cut-site intersected RRBS data, which was merged with the ComGar.WGBS data to provide *Tissue* windows as a negative control.

Genome properties and *cis*-genetic variation

We examined broad-scale relationships between DNA methylation divergence and its chromosomal substrate (population genetic variation, genomic annotation features, chromosome length, positioning along a chromosome, GC-content) primarily using a supervised machine learning regression approach. We complemented this approach with a supervised machine learning classification approach to see if models could capture more variation when only classifying sites into ‘Low’ and ‘High’ levels of divergence. Our reasoning for this approach was to see if discretization of our response variable (DNA methylation divergence) increased the relationships to genome properties. For instance, if DNA methylation divergence operated differently with genome properties at levels of ‘High’ and ‘Low’ divergence instead of strictly along a continuum of divergence values, perhaps the relationship with explanatory variables would be stronger with a binary approach. Our DNA methylation divergence response variable, the binned DMP test-statistic estimates for taxon effects in 5-Kb windows, was transformed into a binary variable corresponding to quantile distributions within each experiment. For instance, within the ComGar.WGBS experiment to create a binary DNA methylation response, we set all methylation divergence values above the 80% quantile to ‘High’, and all windows less than the 80% quantile to ‘Low’. This was repeated for each experiment, corresponding to the distributions and thresholds detailed in Fig S9. Similar covariate permutation importance values were recovered with this approach (Fig S9).

# Supplementary Table Captions (*see .xlsx)*

Table S1. Metadata corresponding to the experimental sampling designs.

Genotype and Hybrid indices were measured in an external study (Knief et al. 2019). ComGar.WGBS indicates the whole genome bisulfite samples.

Table S2. Aligned and deduplicated read pairs and median genome-wide coverage for the 28 male individuals from the resequencing reanalysis.

Table S3. Total read counts, mapping efficiency, and bisulfite-conversion efficiency across the three DNA methylation experiments.

ComGar.WGBS indicates the analyses replicated only within the whole genome bisulfite samples.

Table S4. Determinants of global methylation variation.

Significance of physiological or environmental variables for explaining global patterns in DNA methylation across all three experiments. First, the optimal distance metric was chosen based on Spearman correlations between a data frame of methylation states and explanatory variables using function 'rankindex' in R. Constrained ordinations using vegan were estimated using the supplied distance, with significance of individual variables (or terms) assessed with 10,000 permutations using the 'anova' function. ComGar.WGBS indicates the analyses replicated only within the whole genome bisulfite samples.

Table S5. Overlap between each DNA methylation experiment and the population whole-genome resequencing dataset.

Genetic windows were calculated in 5-Kb genome-wide, and have lower overlap with the RRBS datasets, seemingly because of missing resequencing data in areas of high GC content. ComGar.WGBS indicates the analyses replicated only within the whole genome bisulfite samples.

Table S6. Pairwise correlations between explanatory variables used for supervised machine learning.

Relationships between taxonomic DNA methylation divergence (test-statistic estimates from DMP analyses) were averaged in 5-Kb windows (the resolution of the population genetic data), and were modeled with random forests and boosted regression trees using a regression approach. ComGar.WGBS indicates the analyses replicated only within the whole genome bisulfite samples.

Table S7. Machine learning model fit.

Associations between DNA methylation were assessed with supervised machine learning, and the entire modelling procedure was replicated 3 times providing error estimates. The process was repeated both as a classification problem with a binary response (binned by quantile thresholds), or as a regression problem, using both random forests and boosted trees. The response variable (taxonomic-specific test-statistic from beta-binomial regressions using DSS) was computed within 5-Kb windows either as the mean value (Mean) or the maximum value (Max). ComGar.WGBS indicates the analyses replicated only within the whole genome bisulfite samples.

# Supplementary Figures.


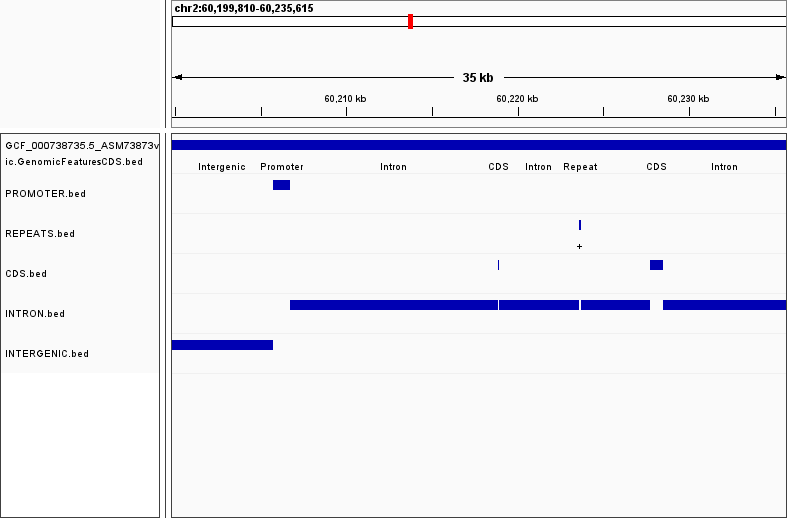


Fig S1. Genomic feature designation.

Example Annotation Feature track for the hooded crow (*C. (c.) cornix*) reference genome. Promoters were identified as CpG islands that overlap the 2-*Kb* region upstream of a gene start. Repeats were identified from RepeatMasker (excluding simple repeats). Genic coding sequencing (CDS) and intronic regions were taken directly from the RefSeq annotation. Remaining regions were annotated as intergenic. Sites were assigned a single genomic context, with priority given in the order as indicated: promoters, repeats, CDS, intron, intergenic. Visualized with the integrative genome browser.


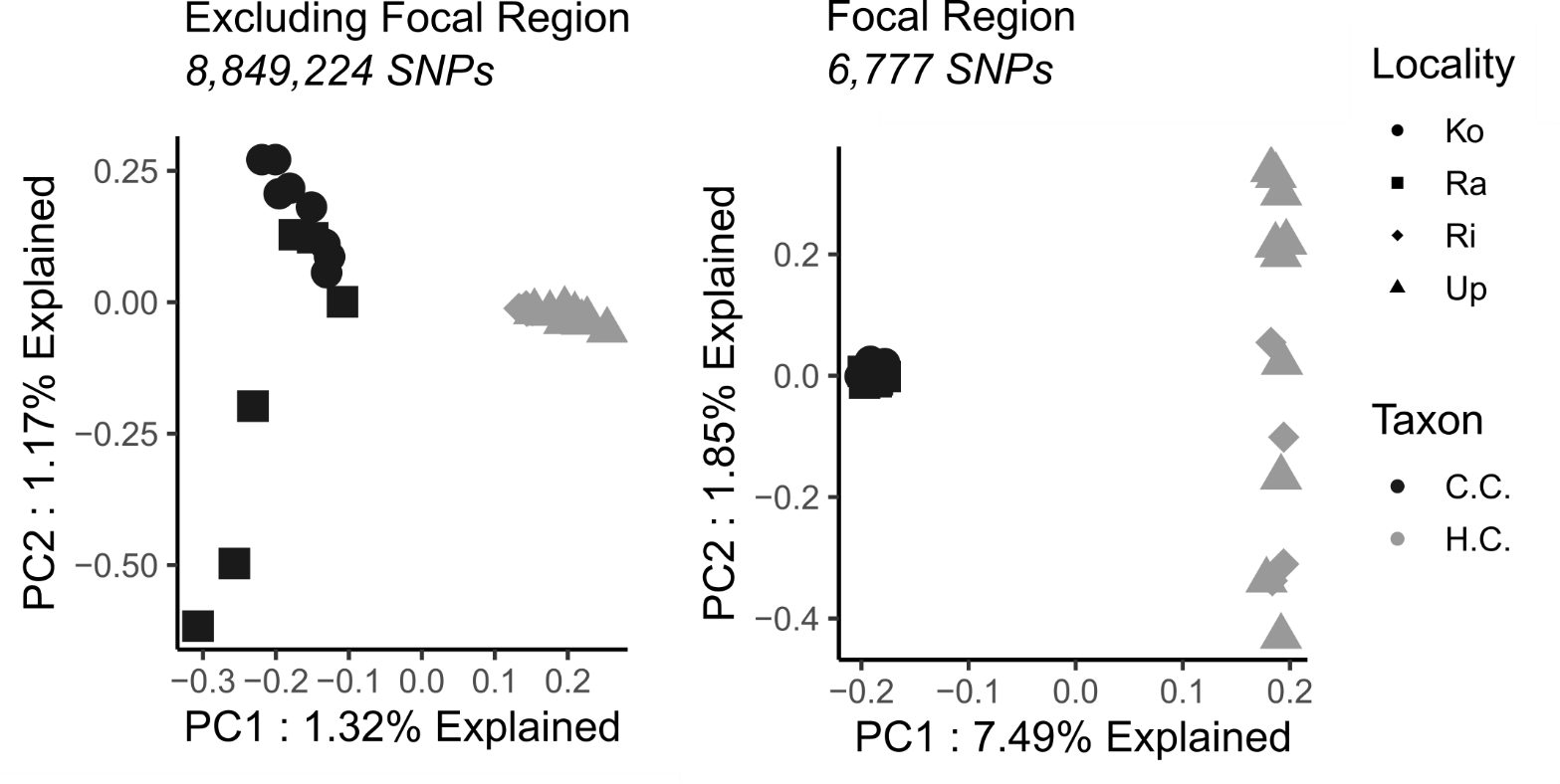


Fig S2. Principal components analysis on genetic whole-genome resequencing data.

Autosomal SNPs excluding the *focal region* on chromosome 18 (left), and on SNPs from only within the focal region on chromosome 18 (right). SNPs were retained if they passed depth and quality filters and provided that at least 90% of individuals had genotypes with at least 3x coverage. Multivariate ordinations on SNPs were analyzed with SNPRelate and visualized with *R* and the *tidyverse*.

Abbreviations: Locality: Ko = Konstanz; Ra = Radolfzell; Ri = Rimbo; Up = Uppsala. Taxon: C.C. = carrion crow (*Corvus (corone) corone*); H.C. = hooded crow (*Corvus (corone) cornix*).


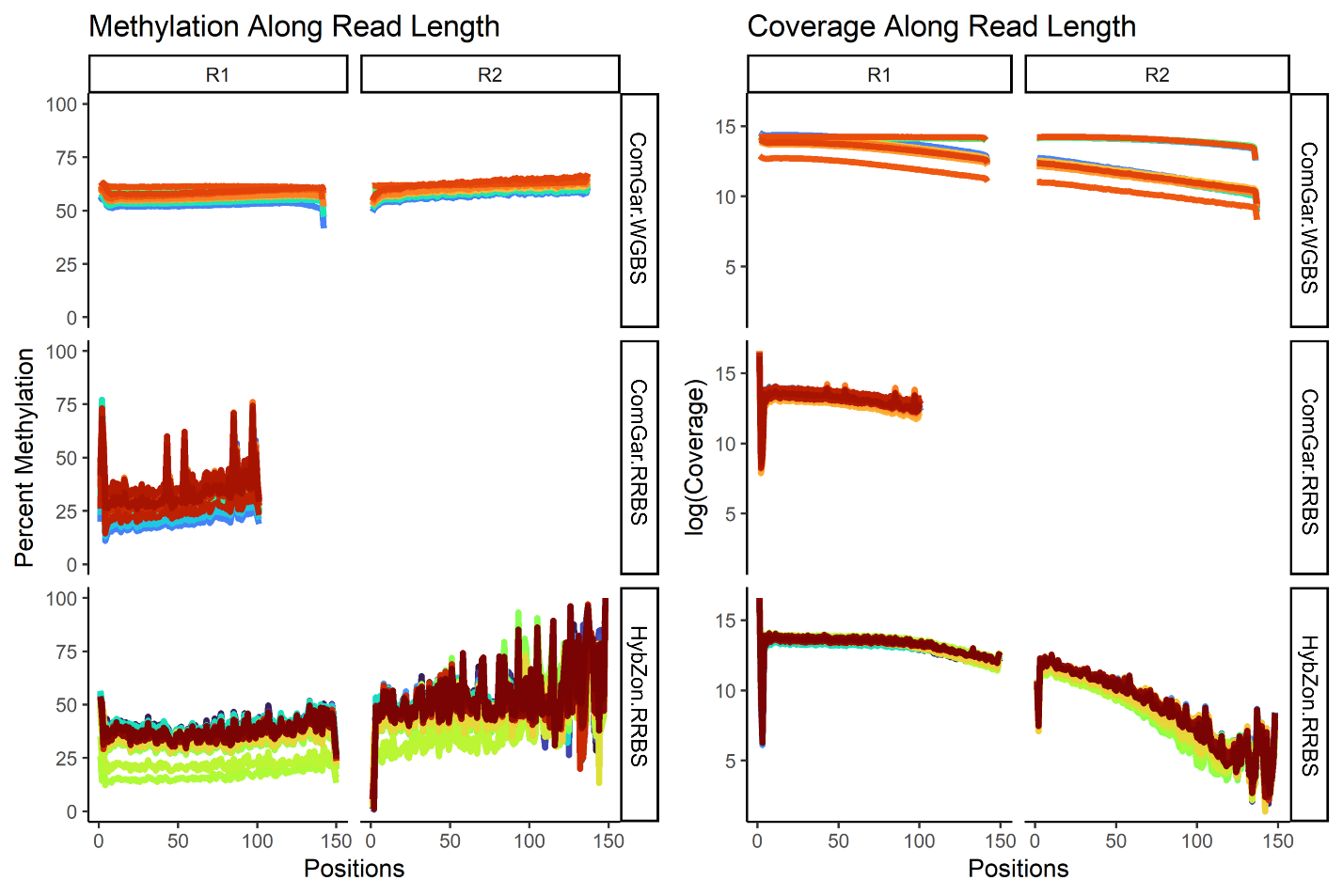


Fig S3. Methylation-bias and coverage along read length for all libraries.

Methylation bias plots showing mean methylation levels and total (log-transformed) coverage across read lengths from the three bisulfite sequencing experiments, with each individual color-coded. Nine bases were trimmed from the R1 and R2 5’ ends of the ComGar.WGBS reads to control for the post-bisulfite adapter tagging artifacts, as well as 1 base from the 3’ end. RRBS reads were trimmed with the ‘—rrbs’ flag of trim_galore. The spikes in RRBS reads appear to arise from high coverage outliers, which are removed in the analysis. The three individuals in the HybZon experiment which exhibit systematic hypomethylation were further analyzed and removed from the analysis due to potential external confounding biological factors. Visualized in *R* with the *tidyverse*.


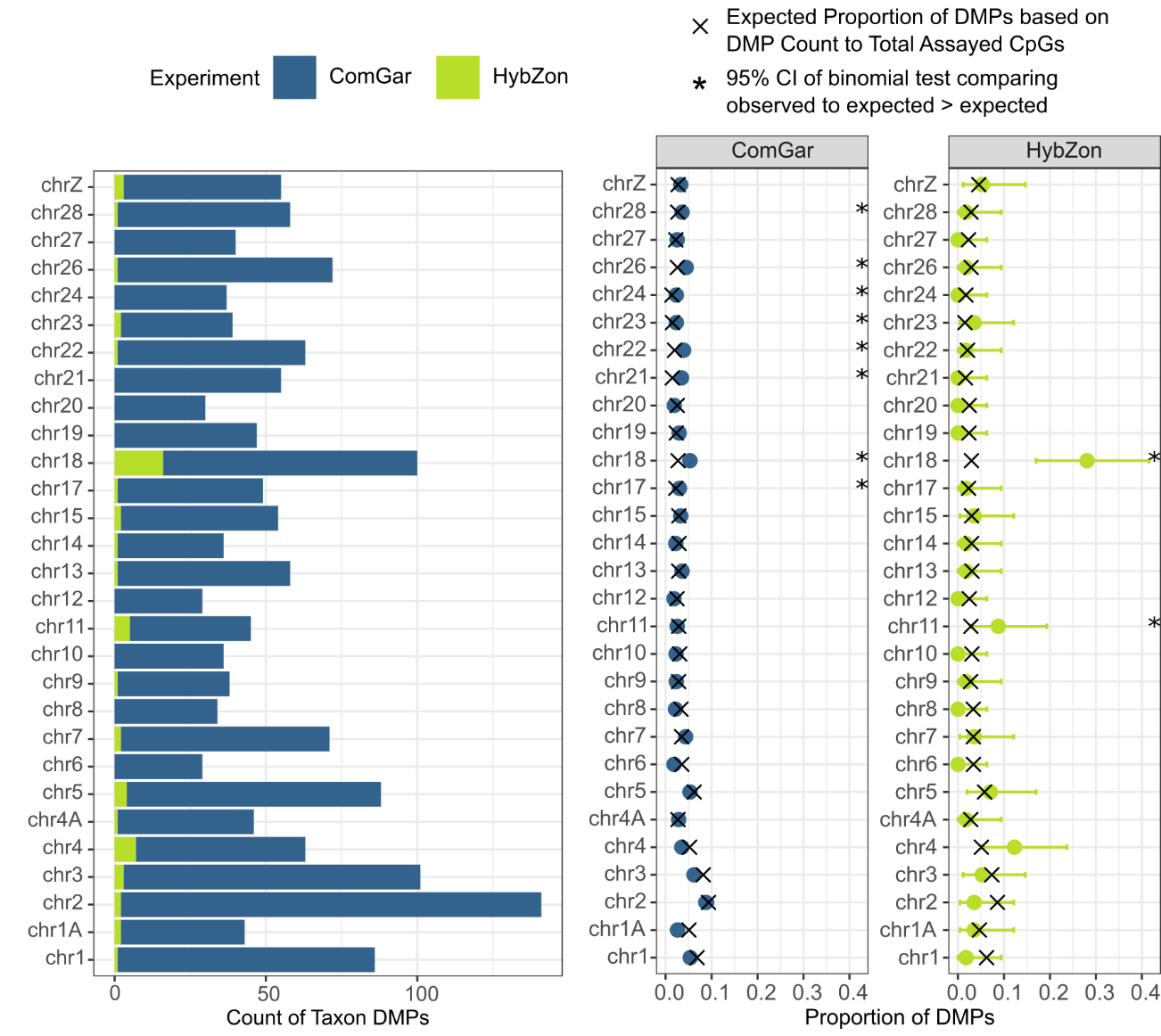


Fig S4. Chromosome-specific enrichment of differentially methylated positions (DMPs).

Counts of taxonomic DMPs across both ComGar and HybZon experiments (left). Observed versus expected taxonomic DMPs for each chromosome (right). Each point represents the observed proportion of DMPs per chromosome, with horizontal error bars showing the 95% confidence interval from a binomial test. The expected proportion of DMPs—based on each chromosome’s total assayed CpGs within each respective experiment—is indicated by the “X”. For each chromosome and experiment, we tested whether the observed number of DMPs exceeded expectations using a binomial test, where the number of successes was the count of observed DMPs, the number of trials was the total number of DMPs in that experiment, and the probability of success was the expected DMP proportion for that chromosome compared to total assayed CpGs. Asterisks (*) denote chromosomes where the lower bound of the 95% confidence interval exceeded the expected proportion, indicating statistically significant enrichment.


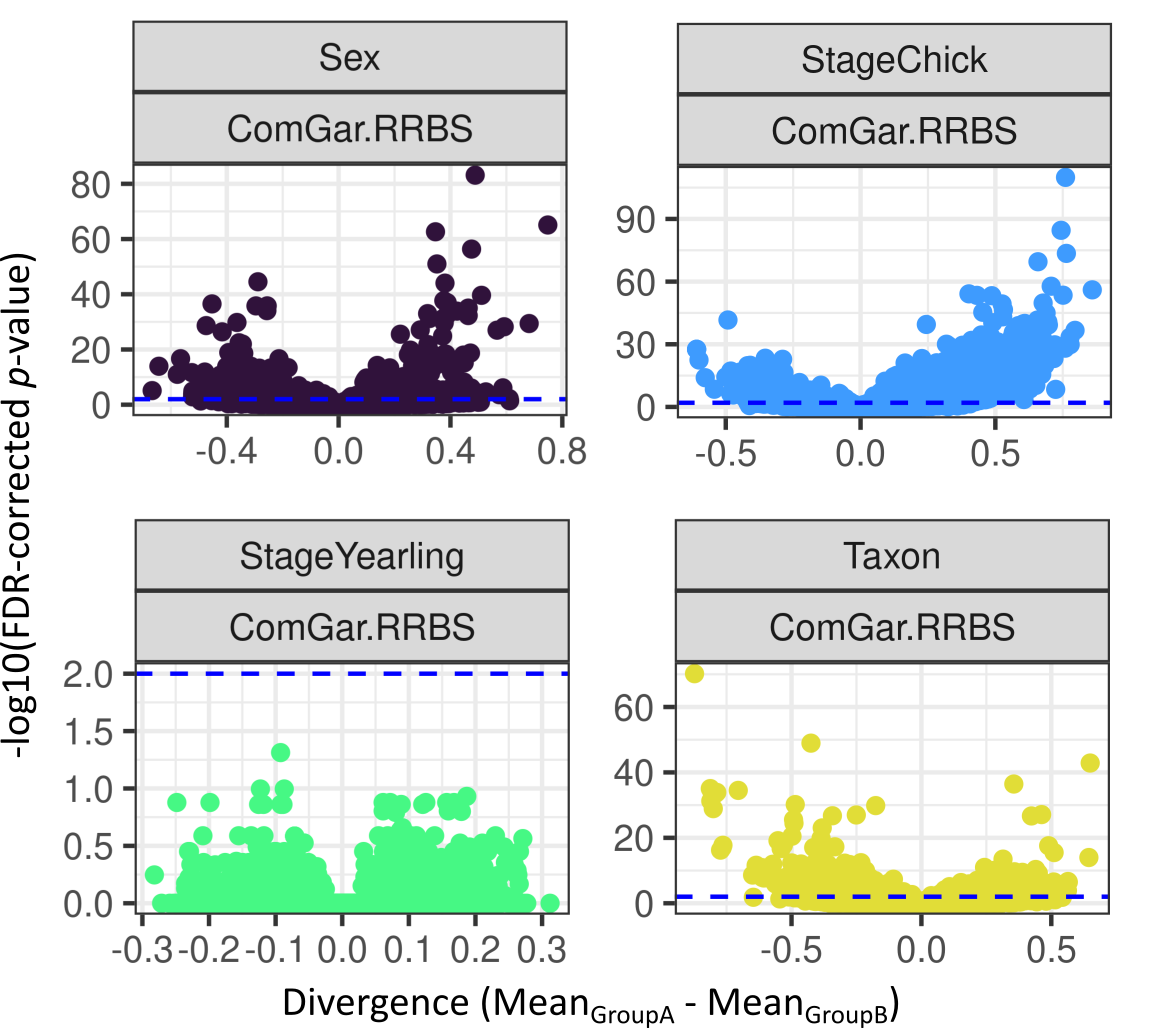


Fig S5. Methylation divergence associated with DMP significance.

DNA methylation FDR-corrected *p*-values from DMP analyses plotted against DNA methylation divergence between groups (ranging from possible -100 to 100% difference) within the ComGar experiment. FDR-corrected *p-*values come from beta-binomial regressions based on read counts from *DSS* (Park & Wu, 2016). Divergence values were calculated by subtracting the mean of each group. FDR thresholds at 0.01 are indicated with the dashed blue line.


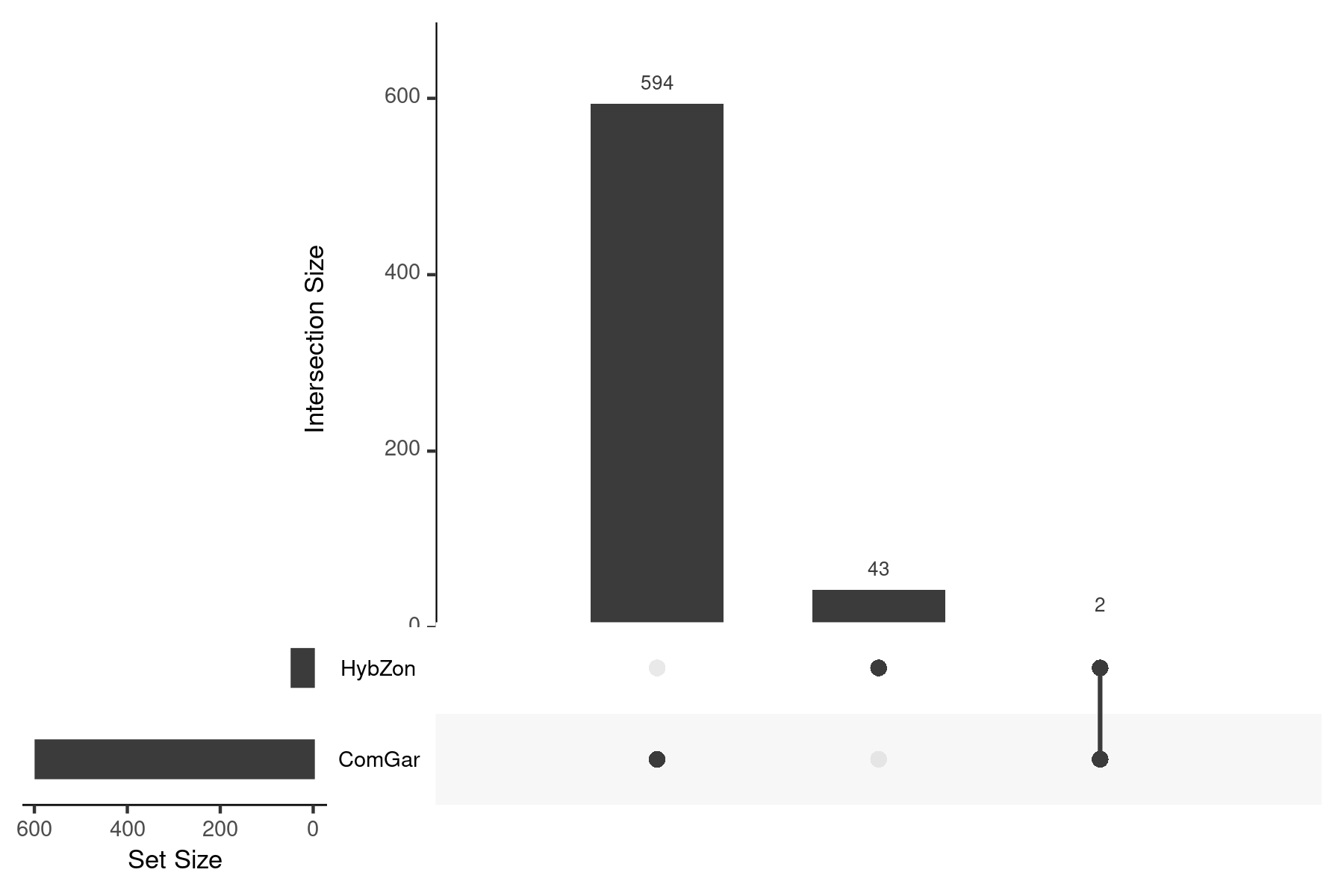


Fig S6. Counts of taxon-related differentially methylated positions and overlap between experiments.

Upset plot showing the distributions and overlap of classified taxon DMPs across experiments. DMPs were classified based on at least 25% difference between groups and an FDR-corrected *p* < 0.01 from beta-binomial regressions, as well as non-significance for other covariates.


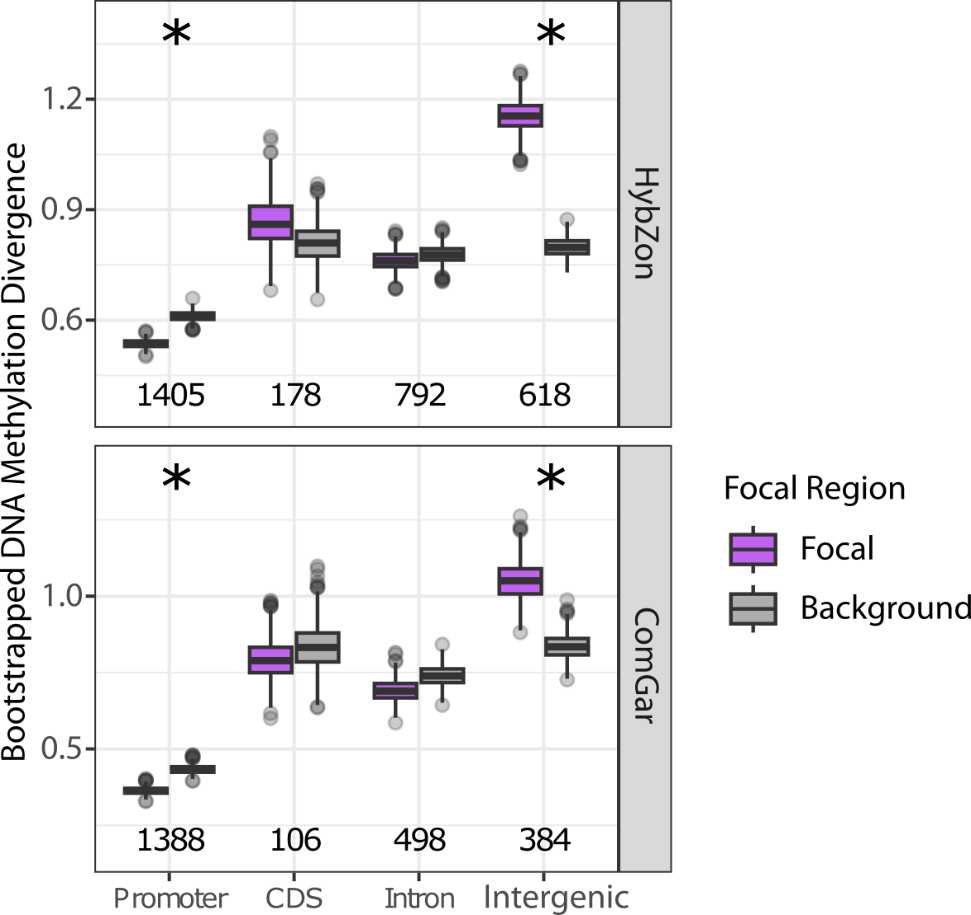


Fig S7. Methylation divergence within and outside the focal region, excluding CpGs surrounding SNPs.

Methylation divergence of CpGs in the *focal region* compared to autosomal background using bootstrap sampling, excluding any CpG positions that are adjacent (1*-Bp* upstream and 1*-Bp* downstream) of any identified SNP in the whole genome resequencing dataset (*n =* 5 HybZon; *n =* 3 ComGar within *focal region*). Boxplots show first and third quantiles, with significance if 95% distribution tails don’t overlap. Total CpGs in the *focal region* indicated below boxplots.


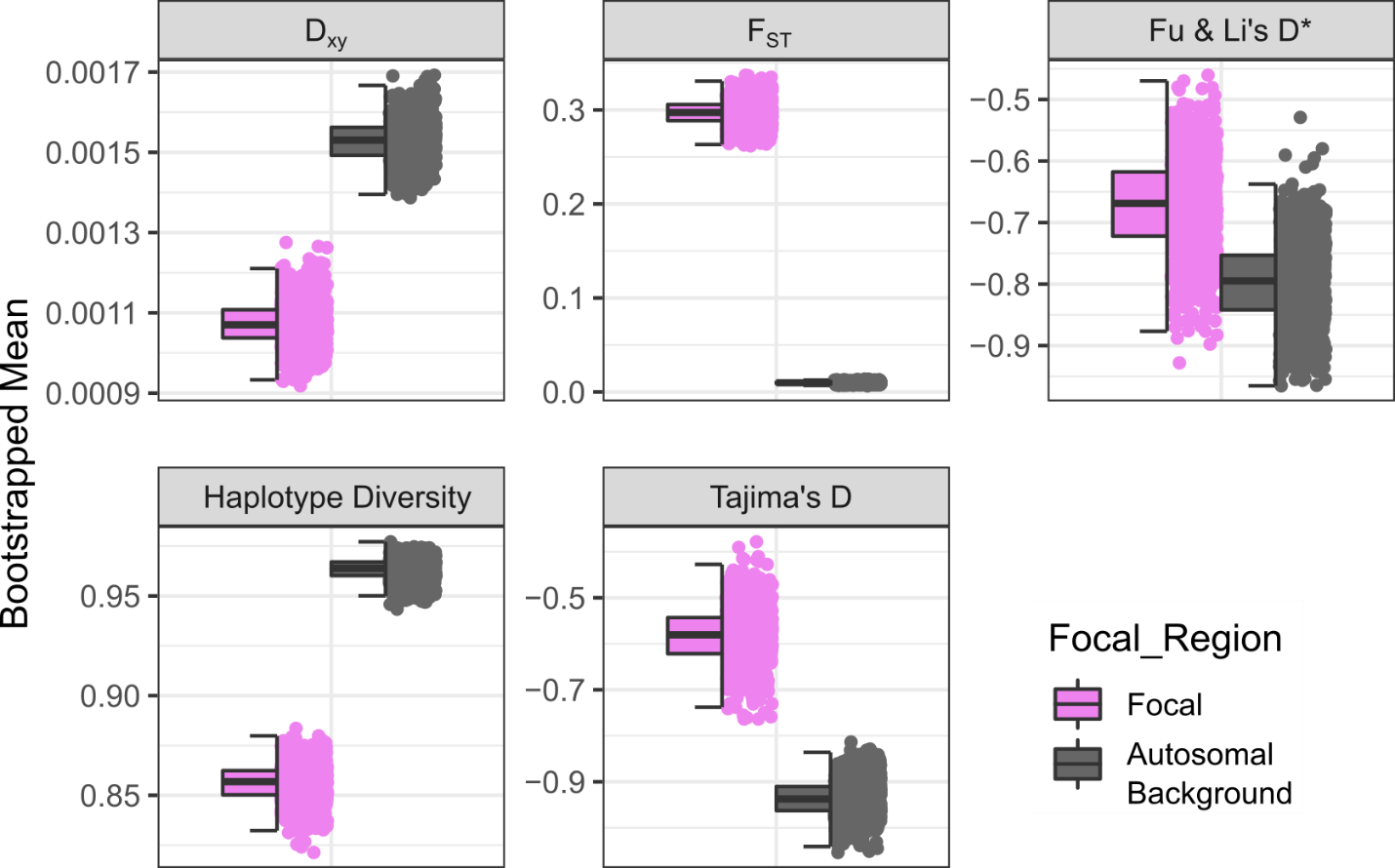


Fig S8. Genetic variation within and outside focal region.

Population genetic variation, calculated in 5-*Kb* windows genome-wide from whole-genome resequencing data. *D_XY_* and *F_ST_* show divergence and differentiation between hooded and carrion crows, while the other metrics show overall variation across all crows. Distributions were created from 1,000 bootstrap sampling events of calculating the mean. Each bootstrap replicate sampled equal numbers of focal and autosomal background windows, which was equal to half the number of total available focal windows.


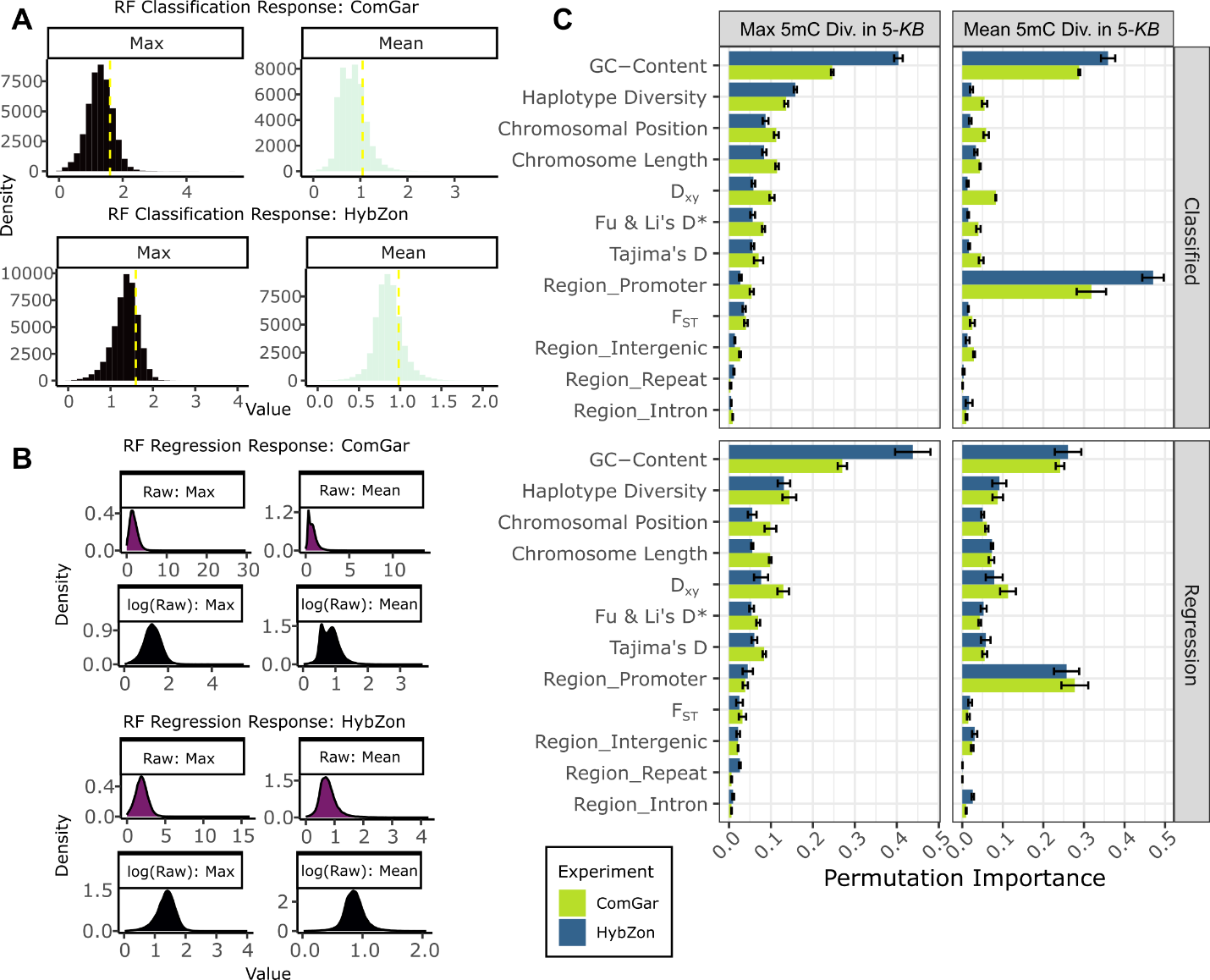


Fig S9. Predicting methylation divergence with chromosomal features.

(**A**) Distributions of taxon-specific methylation divergence (DMP test-statistics) for both experiments with the yellow dashed line indicating the threshold for binary classifications, summarized within 5-*Kb* genomic windows. *Max* used the maximum observed test-statistic observed in the 5-*Kb* window, while *Mean* averaged the test-statistics within the 5-*Kb* window. (**B**) Distributions used for regression modeling, where test-statistics were log-transformed for regression modeling using random forests (RF) and XGBoost. (**C**) Covariate permutation importance from random forest and boosted regression tree models, using both mean and maximum DNA methylation taxonomic test-statistics per window. Cumulative importance scaled to 1.0 per run; models repeated three times for confidence intervals (cf. Fig 4).


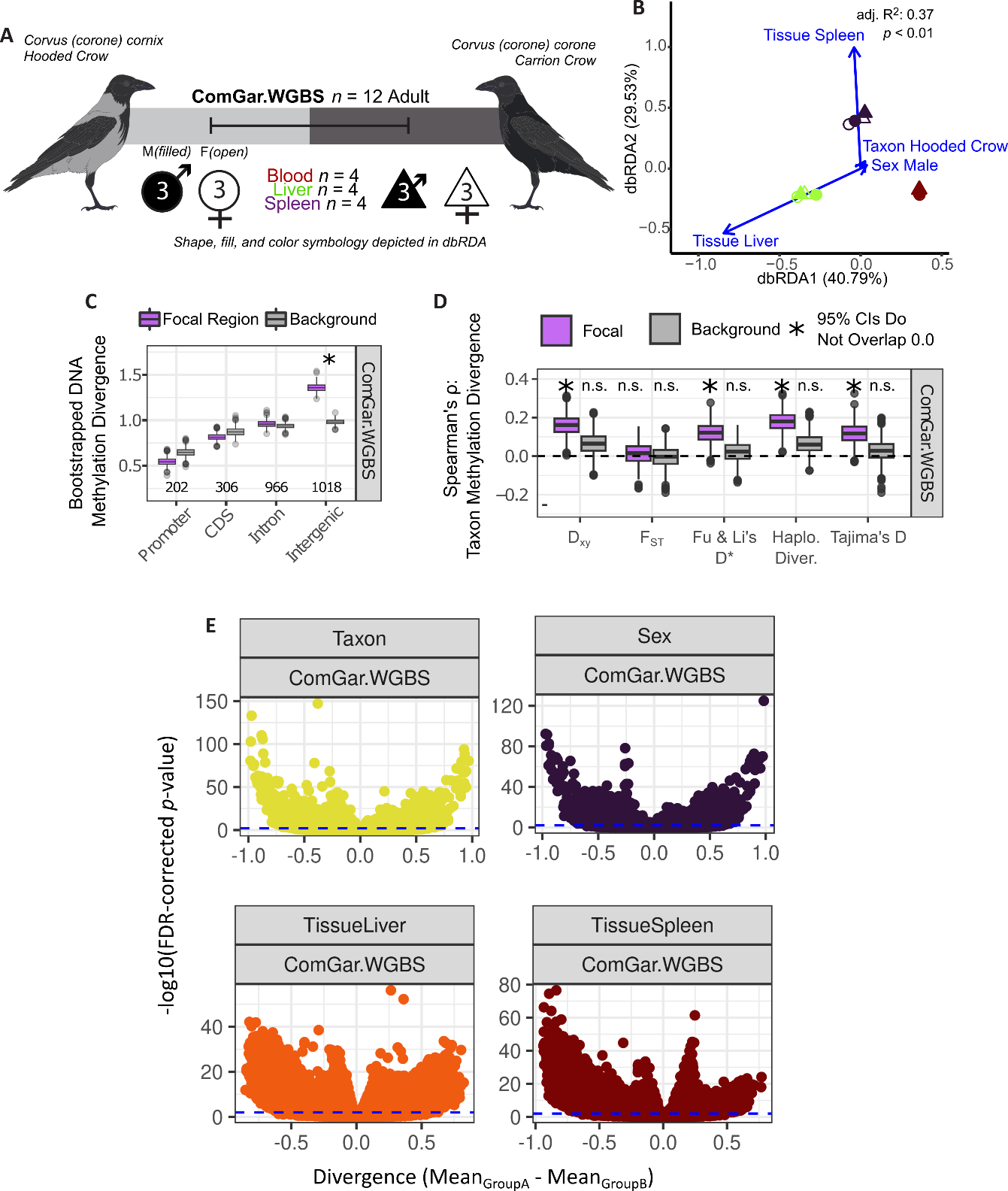


Fig S10. Corroborated analyses using whole genome bisulfite sequencing data.

(**A**) Sampling scheme for whole-genome bisulfite sequencing (WGBS) comprising 12 libraries from 4 individuals (2 *C. (c.) cornix*, 2 *C. (c.) corone*, one male and one female per taxon), each sampled across three tissues. (**B**) dbRDA on methylation profiles (cf. Fig 2). (**C**) Bootstrapped distributions of methylation test statistics from differentially methylated position (DMP) analyses, stratified by genomic region (cf. Fig 3E). (**D**) Bootstrapped Spearman’s rank correlations between pairwise methylation divergence and population genetic differentiation (cf. Fig 4C). (**E**) Relationship between DMP *p*-values and mean methylation differences between focal groups.


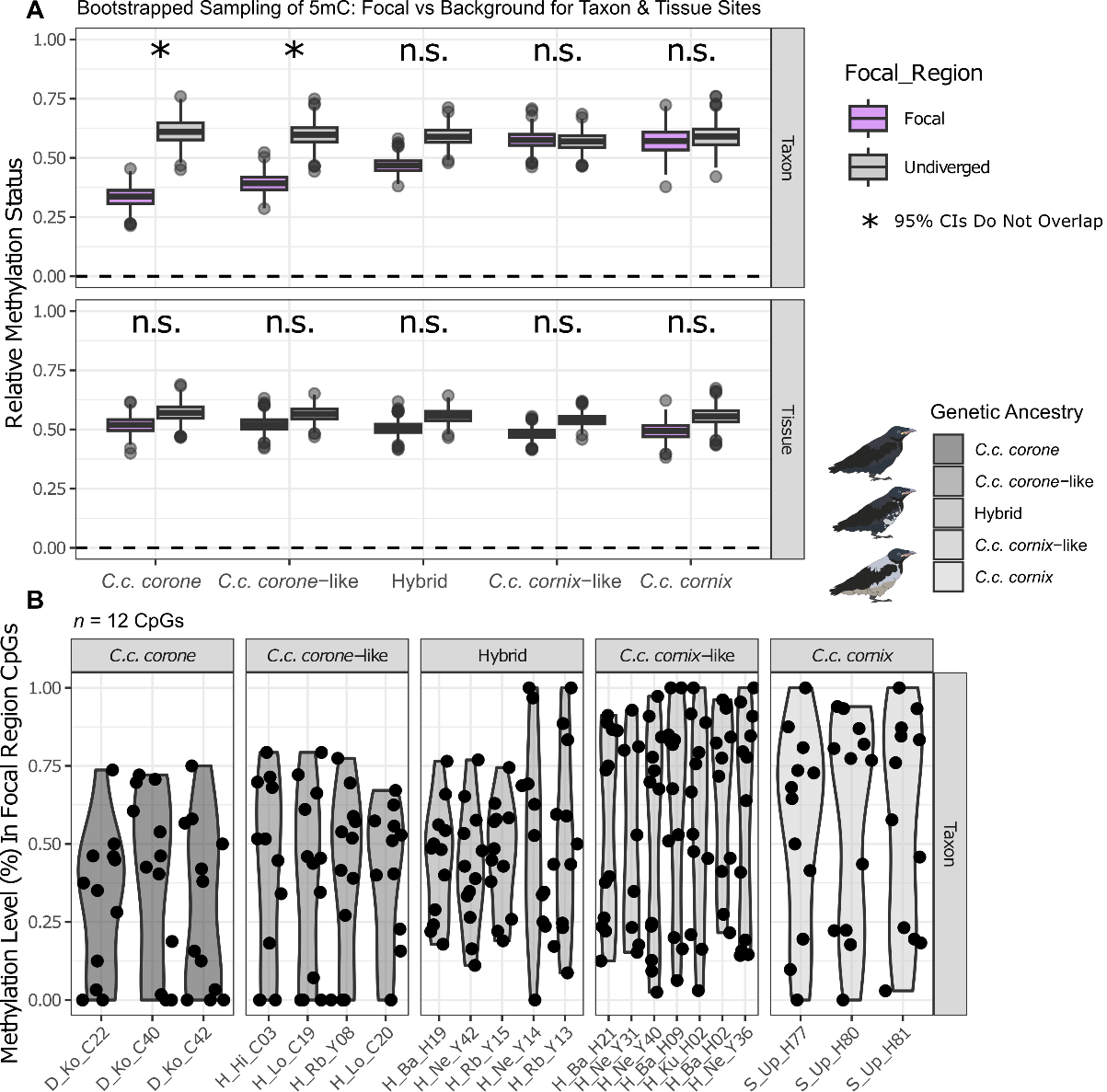


Fig S11. Focal region distributions within the hybrid zone experiment.

First, CpG sites associated with tissue or taxon from the ComGar and ComGar.WGBS experiments were selected based on FDR-corrected *p*-values (*p <* 0.01). Methylation values for hybrids within these sites were examined within the HybZon experiment. (**A**) Differences in methylation levels within the focal region on chromosome 18 versus the autosomal background with bootstrap sampling for tissue and taxon classified sites. We sampled an equal number of autosomal and focal region CpGs equal to the sites in the focal region with replacement and repeated this 1,000 times. Default parameter boxplots show 1^st^ and 3^rd^ quartiles of the bootstrap distributions, significance was indicated if the 95% quartile distributions did not overlap between the focal region and background. Crows within the HybZon are divided by hybrid index. (**B**) Methylation proportions for each taxon-associated CpG *(n* = 12) within the *focal region* for each individual. We observe hypomethylation in *C. (c.) corone* within the focal region and hypermethylation in *C. (c.) cornix*, with hybrids intermediate. Please note that this analysis, which combines ComGar.WGBS with the ComGar RRBS experiment, uses a larger CpG file that is referenced in the supplementary text, and only analyzes RRBS CpGs that fall within a *MspI* cut-site.


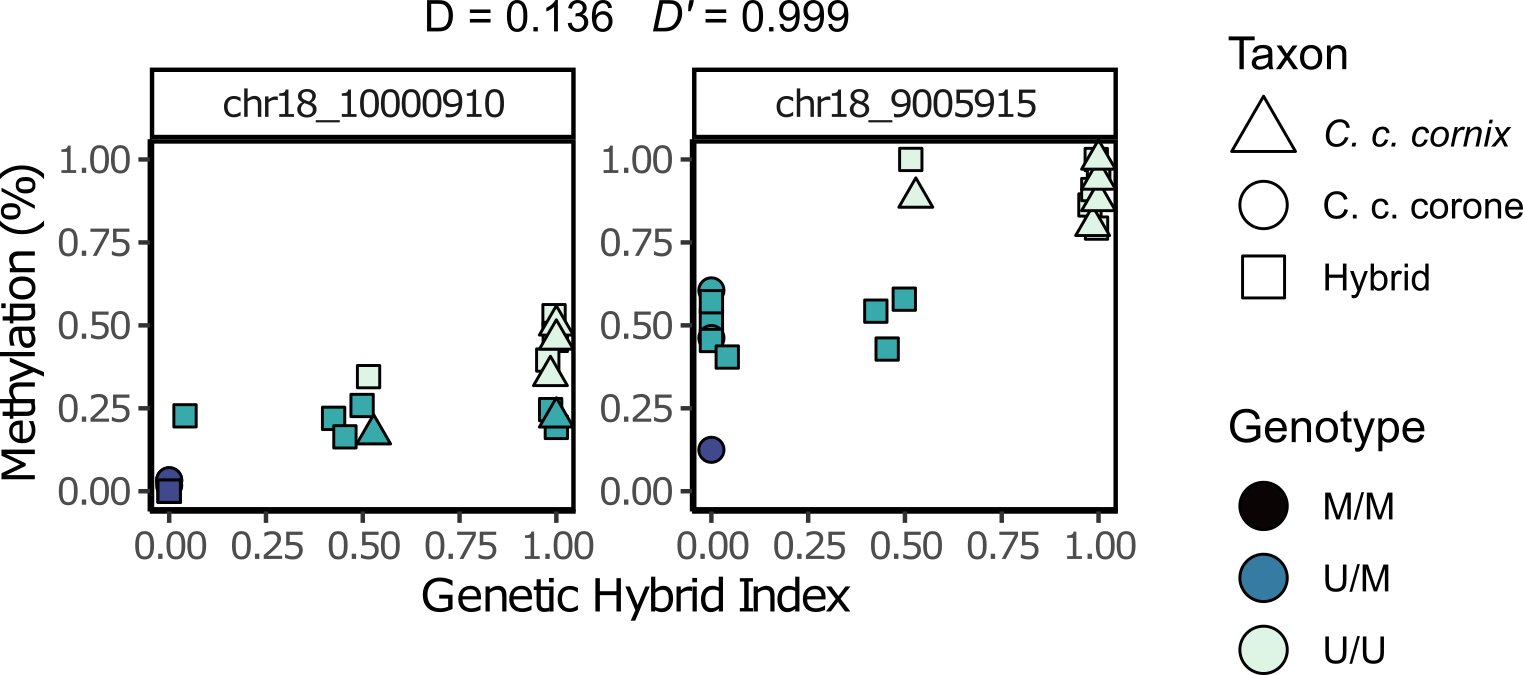


Fig S12. DNA methylation linkage disequilibrium at candidate taxon DMPs.

Taxonomically associated CpGs identified from both the common garden (ComGar) and hybrid zone experiments (HybZon) (*n* = 2). For each site, each individual was assigned a methylation genotype based on k-means clustering of the methylation values (*y*-axis), resulting in the genotypes coloured above (epialleles M and U for methylated and unmethylated). LD between these sites was then calculated using classical genetics approaches.


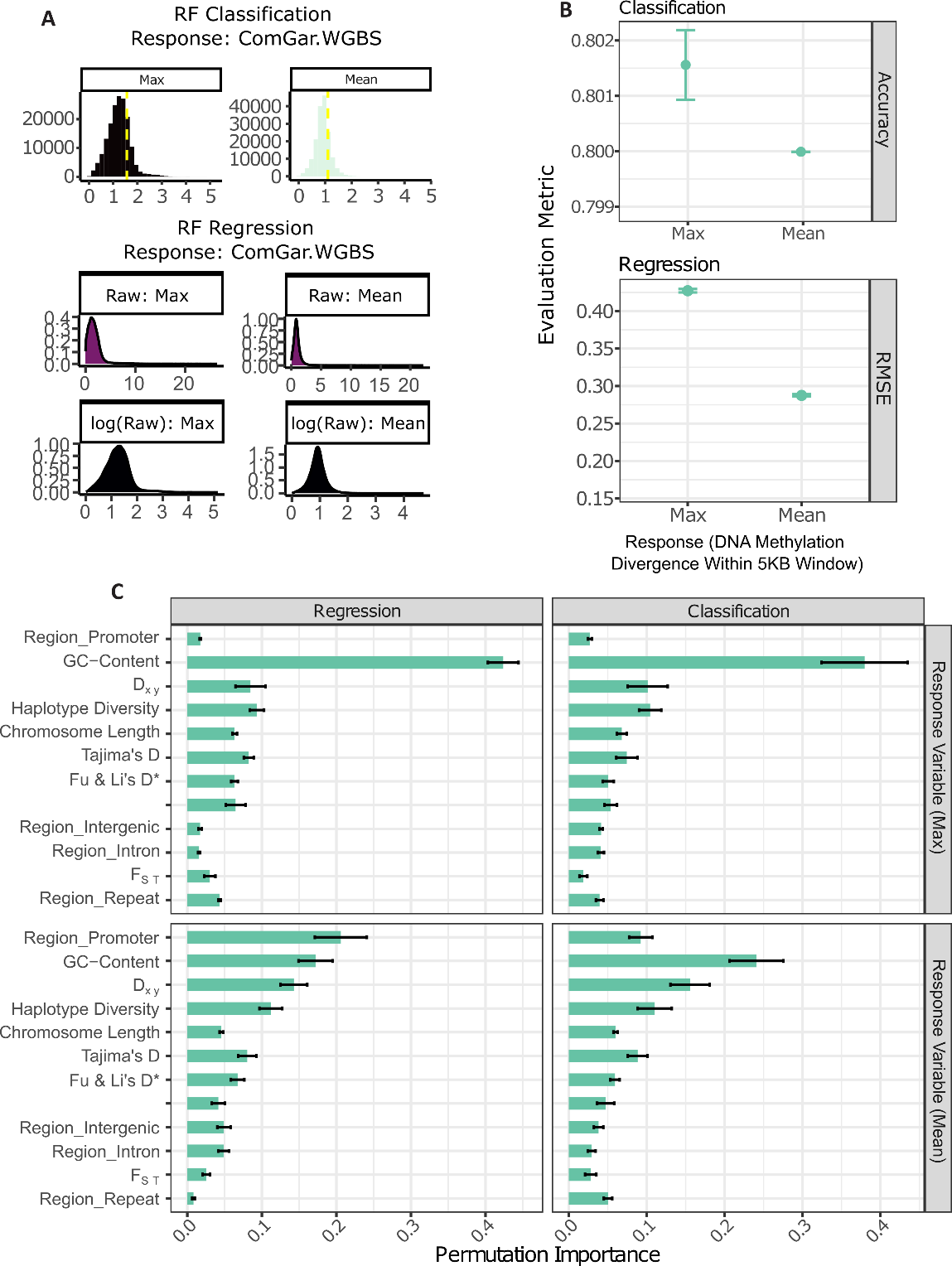


Fig S13. Genomic context of methylation divergence using WGBS.

(**A**) Distributions of taxon-specific methylation divergence (DMP test-statistics) from WGBS data, summarized within 5-*Kb* genomic windows and log-transformed for regression modeling using random forests (RF) and XGBoost. Top panel shows threshold used for binary classification tasks, with the yellow dashed line indicating the cutoff. (**B**) Model fit for classification and regression models, measured with accuracy and root mean squared error (RMSE). (**C**) Covariate permutation importance from random forest and boosted regression tree models, using both mean and maximum DNA methylation taxonomic test-statistics per window. Cumulative importance scaled to 1.0 per run; models repeated three times for confidence intervals (cf. Fig 4).


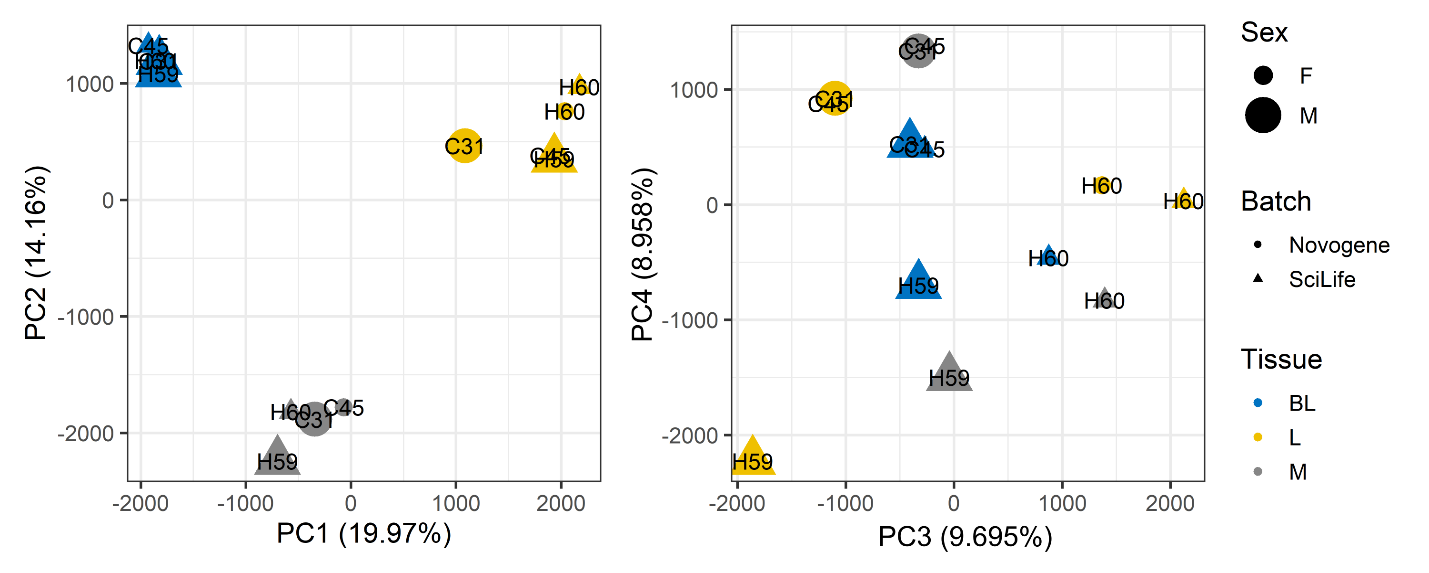


Fig S14. Batch effects for ComGar.WGBS libraries.

Assessing batch effects from the two sequencing centers (Novogene, SciLife) for the ComGar.WGBS dataset, with sexes denoted with size, tissues by color, and shape by sequencing center. Based on the first four axes of a scaled and centered PCA ordination on total DNA methylation data, no batch effects warranted finer scale analysis. Ordinations completed with *prcomp* in *R*.


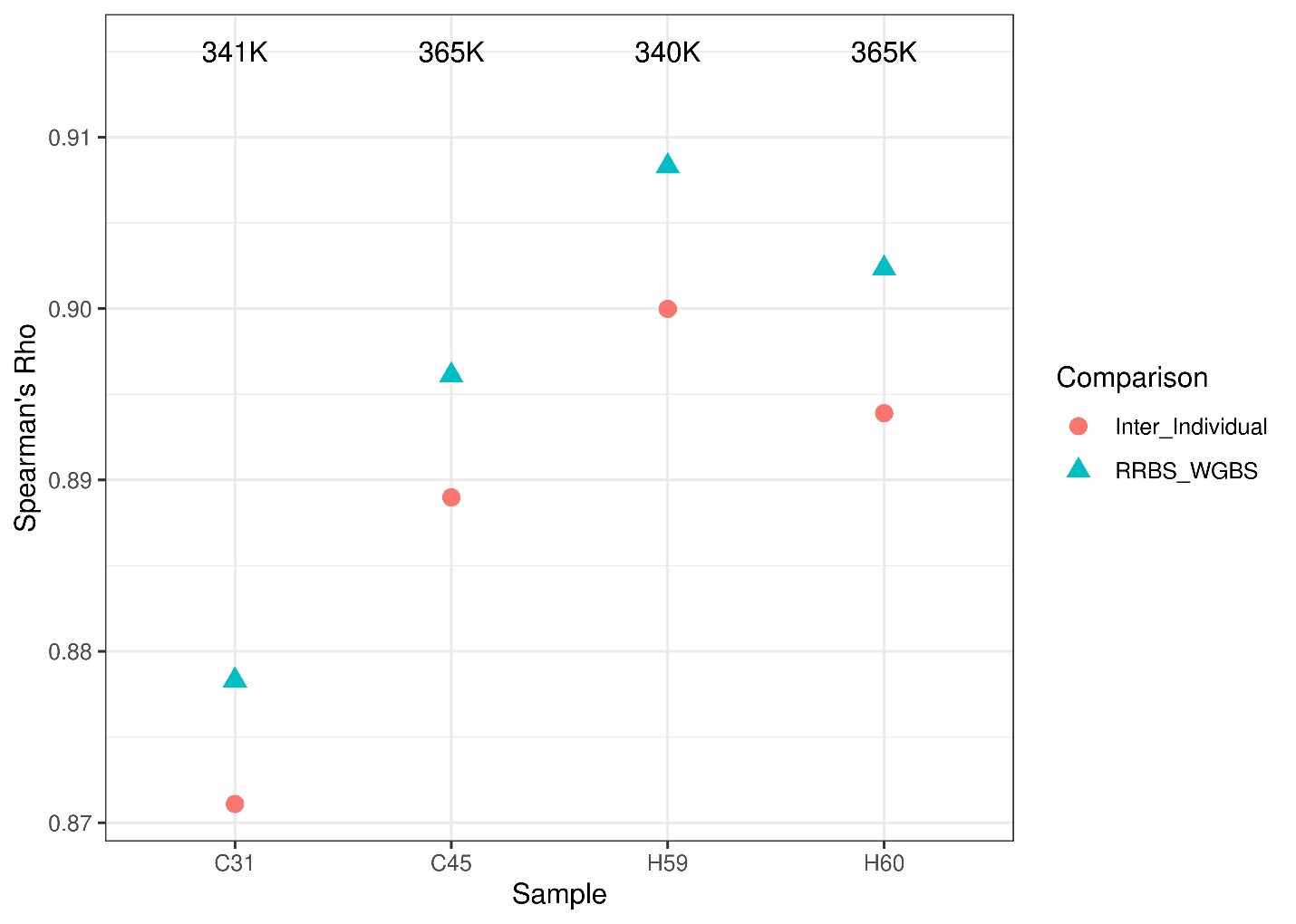


Fig S15. Correlations of DNA methylation between and within individuals.

Spearman’s rank correlations between RRBS and WGBS biological replicates in the ComGar experiment, with a randomly sampled inter-individual correlation for comparison. Labels indicate the number of CpGs considered for the correlation, where each correlation was filtered first to retain only sites with no missing data between the biological replicate and one randomly sampled inter-individual comparison. Correlations were calculated in *R* and plotted with *ggplot2.*


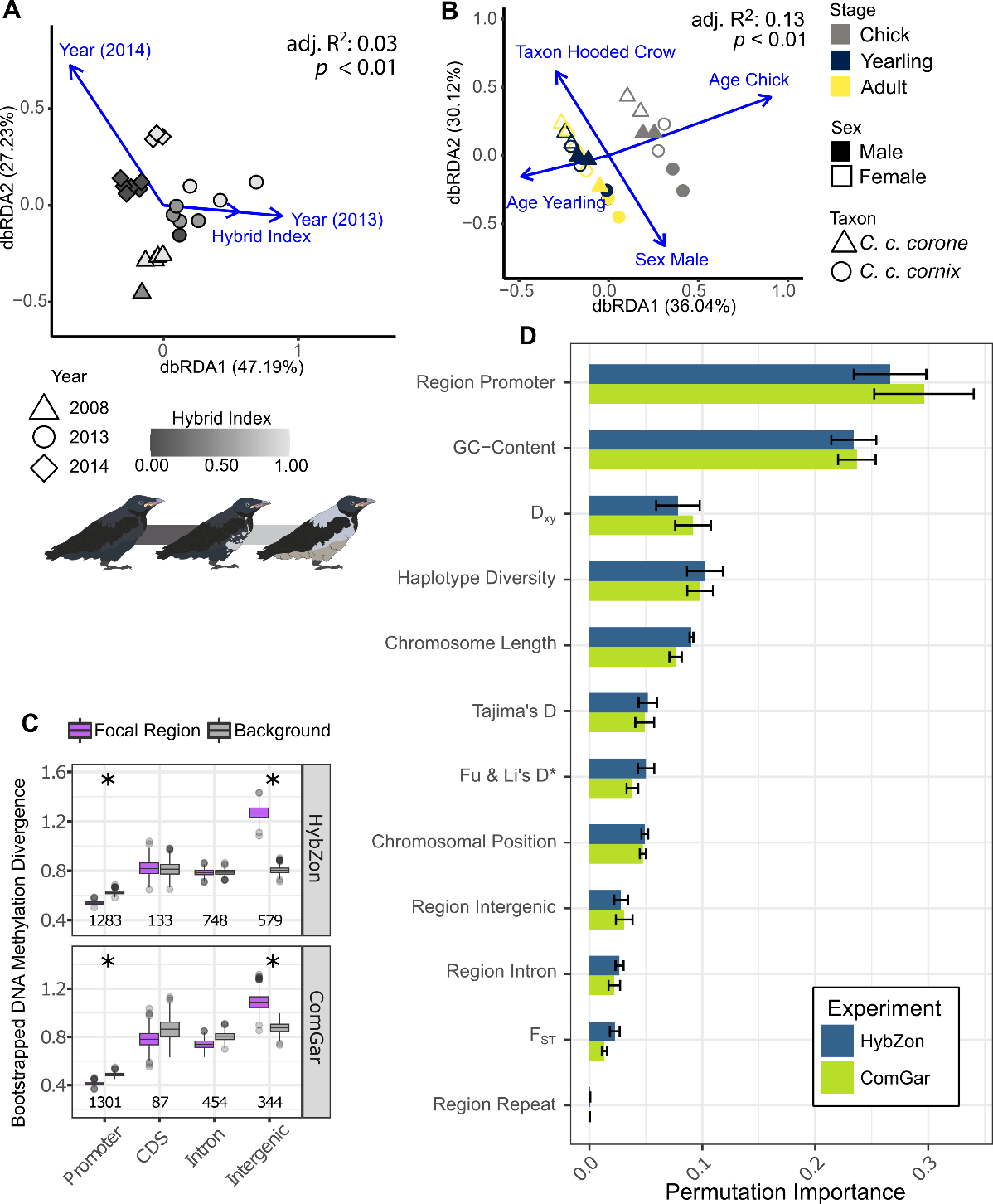


Fig S16. RRBS Analyses repeated for CpGs intersecting *MspI* cut-sites.

Multivariate ordinations (**A, B**), bootstrapped comparisons of *focal region* DMP taxon test-statistics compared to the genomic background (**C**), and machine learning permutation importance for chromosomal substrate (**D**) for RRBS datasets, using a subset of CpGs which directly intersect *MspI* cut-sites (ComGar: 0.700M CpGs, HybZon: 0.834M; see **Supplementary Text**).
